# Determination of the Role of CBP- and p300-Mediated Wnt Signaling on Colonic Cells

**DOI:** 10.2196/resprot.5495

**Published:** 2016-05-13

**Authors:** Michael Bordonaro, Darina Lazarova Lazarova

**Affiliations:** ^1^The Commonwealth Medical CollegeScranton, PAUnited States

**Keywords:** Wnt, butyrate, beta-catenin, CBP, p300, histone acetylation, colorectal cancer

## Abstract

**Background:**

The Wnt signaling pathway, mediated through active beta-catenin, is responsible for initiating the majority of cases of human colorectal cancer (CRC), and we have previously shown that hyperactivation of this pathway by histone deacetylase inhibitors (HDACis), such as butyrate, can induce the death of CRC cells. An important cellular switch that mediates the effects of Wnt-signaling activation is variation in the association between beta-catenin and the transcriptional coactivators cAMP response element binding (CREB) binding protein (CBP) and p300. Association of CBP with beta-catenin is thought to activate a set of genes linked to cell proliferation, while the p300-mediated Wnt genetic program is believed to promote cell differentiation. Small molecule agents have been discovered that modulate CBP/p300 Wnt transcriptional programs by altering the association of CBP and p300 to beta-catenin. ICG-001 and ICG-427 inhibit CBP- and p300-mediated Wnt activity, respectively, while IQ-1 prevents the shift from CBP-mediated to a p300-mediated Wnt activity.

**Objective:**

Aim 1 of this proposal is designed to determine the role of CBP- and p300-mediated Wnt signaling in the response of CRC cells to HDACis. Aim 2 is to determine the role of CBP and p300 in the maintenance of high- and low-Wnt fractions in CRC cell line. Aim 3 will compare the effects of CBP- and p300-mediated Wnt activity on CRC initiation and progression.

**Methods:**

In Aim 1, cells will be cotreated with HDACis and ICG-001, ICG-427, or IQ-1 and the levels of Wnt activity, apoptosis, proliferation, differentiation, and CBP- or p300-beta-catenin binding measured. Aim 2 of this proposal may mirror similar heterogeneity observed in human tumors and which may be of clinical significance. Aim 3 will use CRC cell line model systems of initiation and progression: the normal colon cell lines CCD-841CoN, the adenoma line LT97, the primary colon carcinoma cell line SW480, and the lymph node metastasis cell line SW620. Cells will be treated with HDACis and the small molecule agents, and assayed as described above.

**Results:**

We will also attempt to use changes in CBP- and p300-mediated Wnt signaling to shift colonic cells between cell type, modifying CBP- and p300-mediated gene expression in the LT97 adenoma line to shift the adenoma phenotype to more characteristic of the CCD-841CoN normal cells, or the SW480 carcinoma cells. We will use microarray analyses to determine the patterns of gene expression responsible for these CBP- or p300-mediated changes in colonic neoplastic phenotype.

**Conclusions:**

The findings generated from this study will lead to future, more in-depth projects to further dissect the action of CBP/p300 Wnt–mediated transcriptional programs in colonic neoplasia, with an emphasis on methods to modulate these genetic programs for chemopreventive effect.

## Introduction

Colorectal cancers (CRCs) are amenable to a high degree of prevention [1-12] through diet; however, CRC remains the second leading cause of cancer mortality in the United States. Two large clinical studies convincingly demonstrated a relationship between fiber intake and reduced risk of CRC [10-12]. The protective action of dietary fiber against colon cancer has been attributed to the fermentation of fiber by anaerobic bacteria in the colon, producing butyrate [13], an inhibitor of histone deacetylases (HDACi) that is present in the colonic lumen at high concentrations [14] and has the potential to produce cell cycle arrest, differentiation, and/or apoptosis of CRC cells [15-18].

In addition to butyrate, other HDACis have emerged as promising anticancer agents due to their ability to preferentially induce growth arrest, differentiation, and apoptosis in malignant cells [19,20]. Thus, several HDACis are currently in clinical trial and, recently, the US Food and Drug Administration (FDA) approved the HDACi vorinostat, suberoylanilide hydroxamic acid (SAHA), for the treatment of cutaneous T-cell lymphoma. Considering the increasing clinical evaluations of HDACis, knowledge of how these agents express their antineoplastic properties is important. Initially, the major activity of HDACis was believed to involve net histone acetylation, leading to modified chromatin assembly and altered gene expression [19,20]. Thus, the HDACis sodium butyrate (NaB) and trichostatin A (TSA), which induce apoptosis in SW620 CRC cells in vitro, hyperactivate Wnt transcriptional activity in these cells [21].

Wnt signaling is induced by the binding of Wnt ligands to their cell surface receptors, resulting in inhibition of glycogen synthase kinase-3 beta (GSK-3beta) activity [22,23]. When active, GSK-3beta, in complex with adenomatous polyposis coli (APC) and Axin, promotes the phosphorylation and degradation of beta-catenin [24-26]; however, when GSK-3beta activity is inhibited, dephosphorylated beta-catenin accumulates and interacts with transcription factor/lymphoid enhancer-binding factor (Tcf/Lef) DNA binding proteins [27-32]. Beta-catenin-Tcf (BCT) transcriptional complexes are detected by their ability to drive transcription from Tcf/Lef site-containing promoter constructs [29,30]. It has been established that constitutively activated Wnt signaling, due to mutations in the APC [29-31] and beta-catenin [30] genes, promotes cell proliferation and tumorigenesis in the colon. Thus, the finding that the HDACis NaB and TSA induce programmed cell death, as well as hyperactivate Wnt signaling in SW620 CRC cells [21], seems to be a paradox. This initial observation was subsequently confirmed in a more comprehensive study, which established a linear relationship between the induction of Wnt transcriptional activity and the levels of apoptosis and the inhibition of clonal growth in 10 human CRC cell lines treated with NaB [1]. In addition to butyrate, other structurally unrelated HDACis, including TSA, vorinostat, and MS275 also hyperactivate Wnt activity in CRC cells at concentrations that result in apoptotic levels similar to those induced by a physiologically relevant concentration (5 mM) of NaB [2]. The causative relationship between induction of Wnt activity and apoptosis was analyzed in NaB-treated CRC cells expressing dominant negative Tcf4 (DN-Tcf4), an amino terminally truncated form of Tcf4, which does not bind beta-catenin and which inhibits the activity of endogenous BCT complexes. DN-Tcf4 suppressed the induction of Wnt transcriptional activity by NaB and resulted in reduced levels of apoptosis [1].

While the majority of published studies associate the activation of Wnt signaling with proliferation and tumorigenesis, several in vivo studies [33-36] support the relationship between upregulated Wnt activity and high levels of apoptosis in CRC cells exposed to NaB [1] including: (1) homozygous mutation in the Drosophila homolog of *APC* results in neuronal cell apoptosis in the Drosophila retina [33], (2) expression of stabile, amino-terminally truncated beta-catenin results in 3- to 4-fold higher apoptotic levels in the intestinal villi of transgenic mice [34], (3) conditional targeting of *Apc*, the mouse version of *APC*, in murine neural crest cells results in massive apoptosis of cephalic and cardiac neural crest cells at 11.5 days post coitum [35], and (4) expression of constitutively active beta-catenin in 129/Sv cells of chimeric mice results in apoptosis of these cells [36]. These findings contrast with the reports that decreased Wnt activity, produced by expression of wild-type APC in *APC*-/- CRC cells, induces apoptosis [37,38], probably by downregulation of survivin [39], and that increased levels of beta-catenin protect cells from suspension-induced apoptosis [40]. These contradictory findings can be reconciled by the fact that different levels of Wnt activity are achieved in different experimental systems. Thus, Wong et al [39] proposed that cells exposed to high levels of Wnt activity undergo apoptosis; whereas, cells exposed to moderate levels of Wnt activity maintain a proliferative state, and cells exposed to low levels of Wnt activity undergo differentiation (terminal differentiation followed by apoptosis). The “just right hypothesis” for CRC formation is based upon a similar concept: APC mutations that result in moderate levels of Wnt signaling are optimal for tumor formation and growth; whereas, APC mutations that lead to relatively high levels of Wnt signaling are not selected, most probably due to apoptosis of cells with such mutations [41].

**Figure 1:**
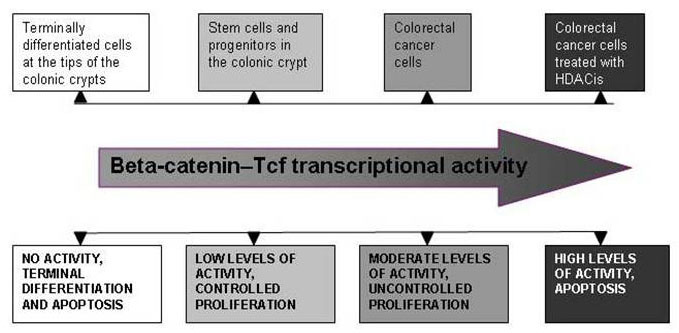
Wnt activity viewed as a gradient. Absence of detectable Wnt signaling, such as in cells at the top of the colonic crypt, results in terminal differentiation, and apoptosis; relatively low levels of signaling, such as in the stem cell compartment of the colonic crypt, lead to controlled self-renewal; moderate levels of signaling, such as in CRC cells, promote proliferation; and relatively high levels of Wnt activity, such as in CRC cells treated with HDACis, lead to enhanced apoptosis. Reproduced from Bordonaro et al [42].

Based upon published studies by others [33-36], as well as by our findings [1,2,21], we postulate that both relatively high and relatively low levels of Wnt transcriptional activity lead to CRC cell apoptosis. Therefore, Wnt activity can be viewed and analyzed as a gradient, within which absence of detectable Wnt signaling (such as in cells at the top of the colonic crypt) results in terminal differentiation and apoptosis, relatively low levels of signaling (such as in the stem cell compartment of the colonic crypt) lead to controlled self-renewal, moderate levels of signaling (such as in CRC cells) promote proliferation, and relatively high levels of Wnt activity (such as in CRC cells treated with HDACis) lead to enhanced apoptosis (Figure 1). Because of mutation, most CRC and/or CRC precursor cells exhibit aberrant Wnt signaling [29-31]. Therefore, these cells undergo apoptosis with relatively high levels of Wnt activity at the right end of the Wnt signaling continuum; whereas, normal colonic cells undergo apoptosis predominantly through regulatory mechanisms that likely lead to terminal differentiation and are associated with the downregulation of Wnt activity (Figure 1).

Based upon our findings in vitro [1,2,21], we propose that high levels of dietary butyrate in the colonic lumen have a “surveillance” function, whereby butyrate drives premalignant and malignant cells with constitutively activated aberrant Wnt signaling into apoptosis through the hyperinduction of Wnt transcriptional activity. The constitutive activation of Wnt signaling makes cells particularly vulnerable to the apoptosis inducing effects of butyrate.

We have established that the hyperactivation of Wnt signaling activity in NaB-treated CRC cells results from increased levels of Ser-37/Thr-41-dephosphorylated (active) beta-catenin, augmented formation of BCT complexes, as well as enhanced BCT complex-DNA binding [1,2]. The HDACis TSA, SAHA, and MS-275 hyperinduce Wnt activity in CRC cells via the same mechanisms [2]. The increased dephosphorylation of beta-catenin [42-44] in the presence of HDACis is triggered at the plasma membrane level. Thus, we demonstrated that Dkk-1 and sFRP2, Wnt-signaling antagonists, which interfere with Wnt ligand-receptor interactions at the cell membrane [45-55], inhibit the upregulation of active beta-catenin and the hyperactivation of Wnt transcriptional activity by the HDACis NaB, TSA, SAHA, and MS-275 in CRC cells [2]. Consistent with this, other groups have provided evidence for autocrine Wnt signaling in CRC cells [52,53].

Results from our in vitro studies [1,2] suggest that the chemopreventive action of HDACis may differ depending upon the levels of Wnt signaling induced in different cancer subtypes. Thus, we have identified two classes of CRC cell lines: those that respond to butyrate treatment with a high-fold induction of Wnt activity and apoptosis (HWA), and those that exhibit a low-fold induction of Wnt activity and apoptosis (LWA) [1]. If such differences exist in vivo, colorectal adenomas, CRCs, and other Wnt signaling-positive neoplasms can be divided into HWA and LWA groups that differ in their response to HDACis, including butyrate derived from dietary fiber. In addition to differences in Wnt activity between different CRC cell lines (ie, HWA vs LWA), variation in basal and HDACi-induced levels of Wnt activity exist between cells of the same CRC cell line. Applying novel flow cytometry-based methodology, we distinguished between cells with high Wnt activity from cells with low or no Wnt activity within individual CRC cell lines [1]. In these experiments, transfections with enhanced green fluorescent protein (EGFP)-expressing vectors under the transcriptional control of a Wnt-sensitive promoter allowed us to evaluate the number of cells with Wnt activity in the cellular population before and after NaB treatment. In both HWA and LWA cell lines, NaB induced Wnt activity in cells with no detectable Wnt activity; therefore, the fraction of Wnt-positive/high-Wnt cells increases in cell lines exposed to NaB. However, HWA cell lines exhibited a larger fold increase in Wnt-positive cells than LWA cell lines. Thus, treatment of HWA cell lines with NaB resulted in more efficient induction of Wnt in an additional number of cells. Based upon our data, the difference between HWA and LWA cell lines in the upregulation of Wnt activity by HDACis derives from (1) HWA cell lines exhibit a greater increase in the Wnt positive cell fraction than do LWA cells, and (2) the levels of Wnt activity per cell are higher in HWA cells than in LWA cells. Further, we have shown that the low-Wnt fraction of a CRC cell population represents cells more resistant to the proapoptotic effects of NaB [1,2]. The greater the fraction of high-Wnt activity cells, the greater the apoptotic response to HDACis. The heterogeneity observed in the levels of Wnt signaling in different CRC cells in vitro may be analogous to the presence of significant heterogeneity in CRC tumors in vivo. It is plausible that the sensitivity of colorectal neoplasms to HDACis depends upon the relative fraction of HDACi-sensitive cells with high levels of Wnt activity. Therefore, the ability to modulate Wnt signaling within populations of neoplastic cells, possibly through Wnt cofactor activity, may lead to enhanced chemopreventive and therapeutic efficacy of HDACis.

An important transcriptional control point that influences the levels and outcomes of Wnt-signaling activation is the association between beta-catenin and the transcriptional coactivators cAMP response element binding (CREB) binding protein (CBP) and p300 [3-6]. Both CBP and p300 are histone acetyl transferase (HAT) proteins known to influence Wnt activity [3-6,56-59]. The interaction between CBP/p300 and Wnt signaling is complex; these cofactors can either up- or downregulate [56-59] Wnt activity. Both CBP and p300 [56-59] bind to Tcf, and this association possibly also mediates the effects of these proteins on Wnt activity. In addition, acetylation of beta-catenin by p300 enhances formation of BCT complexes, and this may be one mechanism by which HAT factors stimulate Wnt signaling [60]. Knockdown of CBP and p300 levels with small-interfering RNA (siRNA) upregulated Wnt activity in the SW480 CRC cell line, suggesting a repressive function for these factors [58]. However, overexpression of p300 or CBP in SW480 cells could not reverse this activation of Wnt activity and actually further upregulated Wnt signaling [58]. This paradoxical finding is possibly due to a dual role for CBP and p300 in both up and downregulating Wnt activity in a context dependent fashion [58].

Interactions between CBP/p300 and Wnt signaling are optimally dissected using inhibitors known to be specific for CBP-Wnt and p300-Wnt transcriptional programs, the small molecule inhibitors ICG-001, ICG-427, and IQ-1. ICG-001 binds to CBP but not to p300, despite the significant homology between these two proteins [3]. SSW480 CRC cells exhibit significant association of CBP with beta-catenin, but minimal beta-catenin-p300 binding. Treatment of SW480 cells with ICG-001, resulting in ICG-001-CBP binding, downregulates the association between beta-catenin and CBP and upregulates the association between beta-catenin and p300 [3]. This transition from CBP to p300 as the predominant binding partner to beta-catenin results in downregulated TOPFlash Wnt reporter activity and decreased steady-state RNA and protein levels for the Wnt target genes survivin and cyclin D1 [3]. This effect of ICG-001 on CBP-mediated Wnt activity was specific to CBP’s role in Wnt signaling, as ICG-001 did not influence other CBP reporters, including AP-1 and CRE [3]. ICG-001 resulted in cancer cell-specific physiological effects; caspase levels, indicative of apoptotic status, were enhanced in SW480 and HCT-116 CRC cells treated with ICG-001, but unchanged in ICG-001-treated CCD-841CoN normal colonic cells [3]. Further, ICG-001 preferentially reduced cell growth and viability in the cancer cell lines, and a water soluble version of ICG-001 reduced the formation of intestinal neoplasms in the Min mouse model of *APC* mutation initiated CRC, demonstrating preliminary in vivo efficacy of these agents [3]. Thus, the data suggest that ICG-001, by switching beta-catenin binding from CBP to p300, downregulates CBP-dependent Wnt signaling, resulting in enhanced CRC apoptosis. In the context of the Wnt signaling continuum, one proposed action of ICG-001 is stimulation of apoptosis by downregulation of Wnt activity below the levels required for maintained proliferation. Alternatively, downregulation of CBP-mediated Wnt activity stimulates p300-mediated Wnt signaling, resulting in the activation of genes promoting terminal differentiation and apoptosis. Further, it is known that Wnt signaling is important for maintaining the pluripotency of embryonic stem cells (ESCs) [6 and references therein]. Another small molecule, IQ-1, maintained Wnt-dependent ESC pluripotency by blocking the transition from CBP-mediated Wnt activity to p300-mediated Wnt activity [6]. The tools available to modulate CBP/p300 Wnt activity also include the small molecule ICG-427, which selectively inhibits p300-beta-catenin association [4].

One factor that must be considered is the CBP/p300 status of colonic neoplastic cells, which has been associated with microsatellite instability (MSI) phenotypes [61]. While most CRCs are microsatellite stable (MSS) and exhibit chromosome instability, approximately 10% to 15% of CRCs are of the MSI type. With respect to human CRC cell lines, HCT-116, SW48, Lovo, LS174T, and DLD-1 are MSI, while the primary CRC/lymph node metastasis paired cell lines SW480/SW620, derived from the same patient, are commonly used representatives of the more prevalent MSS type. Mutation in p300 and CBP, leading to truncated, unexpressed, and/or nonfunctional proteins is often observed in MSI CRCs and CRC cell lines. HCT-116 cells express p300 truncated distal to the HAT domain; however, HCT-116 cells exhibit both p300 and CBP activity. DLD-1 CRC cells, despite being of the MSI phenotype, express at least normal-sized p300 and CBP proteins. Therefore, HCT-116 and DLD-1 CRC cells represent MSI lines that exhibit CBP and p300 activity; consistent with this, treatment with ICG-001-stimulated apoptosis in HCT-116, but not normal, colonic cells [3].

With respect to mechanism(s) by which HDACis may modulate CBP/p300-mediated Wnt activity, we hypothesize that HDACis (1) result in the hyperacetylation of specific proteins that enhance CBP/p300-Wnt complex formation and activity, (2) alter gene expression and the target genes modulate CBP/p300-mediated Wnt activity, (3) result in a more open chromatin configuration, allowing enhanced access of CBP/p300-Wnt complexes to target DNA promoter/enhance regions, and/or (4) hyperacetylation of histone and nonhistone proteins resulting from HDACi inhibition complements the acetylation induced by the HAT proteins CBP and p300. Thus, treatment with HDACis can enhance either CBP- or p300-mediated Wnt activity. Whether HDACis activate the CBP or p300 pathways would be dependent upon which of those two pathways are most active in the cells at the time of HDACi treatment; thus, in this scenario, the small molecule inhibitors shift the cells toward either CBP-Wnt or p300-Wnt signaling, reinforcing whichever of the two pathways is activated in the cells. There are many possible mechanisms by which HDACis both generally upregulate Wnt activity and apoptosis as well as more specifically influencing CBP/p300-mediated Wnt activity; these mechanisms of action will be explored in a separate study building upon the findings of the current proposal. The specific aims of the current proposal are specifically focused upon using the small molecule inhibitors ICG-001, ICG-427, and IQ-1 to evaluate the requirement for CBP-Wnt and p300-Wnt interactions with respect to the induction of Wnt activity and of apoptosis by HDACis, and tumorigenic progression represented by a cell culture model of colonic neoplasia.

In summary, CBP- and p300-mediated Wnt signaling likely influences the (1) differential response of CRC cell lines to HDACis, (2) maintenance of low- and high-Wnt fractions within single CRC cell lines, which may be representative of in vivo tumor heterogeneity, and (3) progression of colonic neoplasia from normal cells to metastatic carcinoma. In the current proposal, we will use in vitro models of CRC to explore the role of CBP/p300-mediated Wnt activity in these phenomena. The findings will lead to future projects to explore potential chemopreventive and therapeutic approaches based upon the modulation of CBP/p300-mediated Wnt activity. For example, it may be possible to modulate CBP- versus p300-mediated Wnt signaling to make colonic neoplasms more sensitive to the proapoptotic action of HDACis. Thus, pharmacological interventions based upon the small molecule inhibitors ICG-001, ICG-427, and/or IQ-1 can be used with butyrate derived from high fiber diets (chemoprevention) or therapeutic application of vorinostat (treatment and/or chemoprevention) to enhance the antineoplastic properties of these HDACis. If, for example, it is discovered that CBP-mediated Wnt activity promotes CRC cell proliferation and inhibits the apoptotic pathway induced by HDACis, then cotreatment with HDACis and a pharmacological agent similar in action to ICG-001 (CBP-Wnt inhibitor) will have greater antineoplastic efficacy than application of HDACis alone. Further, if, for example, p300-mediated Wnt signaling is shown to mediate the proapoptotic effects of HDAC-induced Wnt hyperactivation, then suppression of CBP-mediated Wnt activity with ICG-001 will enhance the p300-mediated pathway [3]. On the other hand, if findings from the current proposal demonstrate that CBP-mediated Wnt activity is responsible for the enhanced apoptosis promoted by HDACi-induced apoptosis, then pharmacological agents similar to ICG-427 and IQ-1 would be appropriately used in conjunction with HDACis for maximal therapeutic effect. These treatments may be suitable for suppressing tumor initiation (chemoprevention) and/or inhibiting tumor progression (therapeutics). Therefore, future in vivo studies should be directed toward evaluating combinatory treatments of HDACis with modulators of CBP/p300 signaling in mouse models of CRC to determine whether tumor/size incidence is reduced compared with treatment with each class of agent in isolation. If successful, these in vivo studies would lead to the formulation of CBP/p300 Wnt-modulating agents suitable for human use in clinical trials.

## Methods

### Aim 1. Determination of the Role of CBP and p300 in the Response of CRC Cells to Histone Deacetylase Inhibitors (HDACis)

We hypothesize that the effects of HDACis on Wnt signaling and cell fate are mediated by changes in the association of CBP and p300 with beta-catenin, and by modulation of BCT complex formation and Wnt activity. To investigate this hypothesis, we will first investigate the effects of CBP- and p300-mediated Wnt activity in the HWA HCT-116, and DLD-1 CRC cell lines, which respond to HDACis with a high-fold induction of Wnt activity and apoptosis (HCT-116 cells, 21.8-fold induction Wnt, 2.5-fold induction apoptosis; DLD-1 cells, 15.4 Wnt, 3.5 apoptosis), the LWA HT29 cell line, which exhibit a low-fold increase in Wnt activity and apoptosis after HDACi treatment (1.4-fold Wnt, 1.5-fold apoptosis), and SW620 cells, which exhibit a moderate-fold increase in Wnt activity and apoptosis after HDACi treatment. Although there are no clear divisions between HWA and LWA cell groups, based upon findings with 10 CRC cell lines, HWA is defined as a 10-fold or greater increase in Wnt activity and a 2-fold or greater increase in apoptosis; LWA is defined as a 1- to 4-fold increase in Wnt activity and a 1- to 1.5-fold increase in apoptosis, and a moderate fold increase in Wnt activity and apoptosis is between 5- and 10-fold for Wnt activity and 1.5- and 2-fold for apoptosis [1,2]. In general, the highest induction of apoptosis is observed in those CRC cells that respond to HDACis with a 15-fold or greater increase in Wnt activity, and a linear relationship exists between induction of Wnt activity and apoptosis [1,2]. Therefore, we hypothesize that modulation of CBP/p300-mediated Wnt activity can enhance the ability of HDACis to upregulate Wnt activity, resulting in higher levels of apoptosis. Although HCT-116 cells are of the MSI phenotype and express a truncated form of p300 [60], this cell lines is characterized by CBP and p300 HAT activity and has been successfully used to evaluate the effects of ICG-001 on cell apoptosis [61,63,63].

The reported effects of CBP and p300 on Wnt activity are most likely mediated through changes in the association of these HAT proteins with Wnt signaling factors, particularly beta-catenin [3-6]. CBP [59] and p300 [62] may associate with Tcf factors and that this may also influence Wnt activity; in addition, p300 HAT activity has been reported to enhance BCT complex formation through increased acetylation of beta-catenin [59]. Therefore, we will first determine whether treatment with HDACis alters the association of CBP and p300 with beta-catenin through coimmunoprecipitation assays, which will be performed essentially as described [1-3]. Initial HDACi treatment will be with 5 mM sodium butyrate or 10 μM vorinostat for 24 hours, which efficiently upregulates Wnt activity and apoptosis in CRC cells [1,2]. If necessary, we will adjust the concentration of HDACis and exposure time to these agents for these and subsequent experiments. Subsequently, we will also use coimmunoprecipitation to determine the effects of HDACis on the association between CBP or p300 and Tcf1 and Tcf4, and between Tcf factors and beta-catenin.

To determine how CBP- or p300-mediated modulation of Wnt activity specifically influences the effects of HDACis on Wnt activity and cell physiology, CRC cells will be cotreated with HDACis and with ICG-001, ICG-427, or IQ-1 for 24 hours, or with each of the small molecule inhibitors alone. In addition, experiments will be conducted with the combination of ICG-001 and IQ-1. ICG-001 and ICG-427 inhibit CBP- and p300- mediated Wnt activity [3-5], respectively, while IQ-1 prevents the shift from CBP-mediated to p300-mediated Wnt activity [6]. Therefore, the combination of ICG-001 and IQ-1 is specifically designed to knockdown CBP-mediated Wnt activity in the absence of any concomitant upregulation of p300-mediated Wnt activity. Thus, treatment with ICG-001 alone is expected to specifically inhibit CBP-mediated Wnt activity, with the possibility of an upregulation of p300-mediated Wnt activity. Cotreatment with ICG-001 and IQ-1 is expected to suppress CBP-mediated Wnt activity without increasing p300-mediated Wnt activity. Therefore, for all three aims of this proposal, comparison of the effects of ICG-001 alone to that of combinatorial treatment with ICG-001 and IQ-1 will allow us to distinguish cellular effects specifically due to downregulated CBP-mediated Wnt activity alone (combinatorial treatment of ICG-001 and IQ-1) to effects due to downregulation of CBP-mediated Wnt activity coupled to a relative increase in p300 Wnt activity (ICG-001 alone). Dependent upon cellular context, it is also possible that ICG-001 alone will inhibit CBP-mediated Wnt activity without increasing p300-mediated Wnt activity; thus, an increase in p300-beta catenin associated will not be assumed after ICG-001 treatment, but carefully assayed by coimmunoprecipitation experiments. In summary, the three small molecule inhibitors, used alone and in combination, are effective tools to dissect the relationships between CBP- and/or p300- mediated Wnt activity, cell physiology, and effects of HDACis.

The appropriate levels of the small molecule effectors will be determined empirically. After treatment with these combinations of agents, coimmunoprecipitation assays, performed as described [1-3], will be used to determine CBP/p300 binding to beta-catenin, Tcf1, and Tcf4, as well as the association between beta-catenin and Tcf1 and Tcf4. Measurements of Wnt transcriptional activity in CRC cells treated with these agents will be performed with the Wnt reporter TOP/FOP luciferase reporter system that has wild-type (TOP) or mutant (FOP) Tcf binding sites upstream of a minimal c-fos promoter [29,30]. The ratio of luciferase expression driven by the wild-type TOP promoter to that driven by the mutant FOP promoter specifically assays the contribution of Wnt activity to the expression of luciferase [1,2,21,29,30]. Wnt-driven luciferase expression will be measured using the dual luciferase assay system. For these transfections, we will use Geneporter or Lipofectamine 2000 as described [1,2]; alternatively, for higher transfection efficiencies, the Nucleofector system will be used. We will measure the expression of the Wnt target genes survivin, cyclinD1, and c-myc, which are important mediators of decisions of cell proliferation and apoptosis. ICG-001 has been shown to downregulate the expression of survivin and cyclin D1 in CRC cells [3]; all three genes are downregulated by butyrate [64-66].

Further, expression of the receptor tyrosine kinases EphB2 and EphB4, which binds to EphrinB2, is controlled by CBP- and p300-mediated Wnt signaling, and these differences of expression influence cell physiology [67]. EphB4 is usually absent in normal colonic cells but tends to be expressed in CRC; expression level correlates to more advanced neoplasia [67 and references therein]. On the other hand, EphB2 is expressed by normal colonic progenitor cells and tends to be downregulated in CRC [67 and references therein]. CBP-mediated Wnt signaling, which promotes colonic cell proliferation while inhibiting differentiation [3-5], activates EphB4 expression while inhibiting levels of EphB2 [67]; this is consistent with a possible role for CBP-mediated Wnt signaling in promoting colonic neoplasia [3-5]. Knockdown of EphB4 expression suppresses tumor growth and metastases [67], which is consistent with the observed antitumorigenic activity of the CBP-Wnt inhibitor ICG-001 [3-5]. Thus, expression of EphB2 and EphB4 will be measured in the colonic cell lines exposed to the small molecule inhibitors and HDACis. For all proteins (survivin, cyclinD1, c-myc, EphB2, and EphB4), Western blotting using commercially available antibodies will be used to determine changes in expression levels.

Aspects of CRC cell physiology that may be modulated by CBP/p300-mediated Wnt signaling will be determined. For example, it has been reported that inhibition of CBP-mediated Wnt activity stimulates apoptosis in CRC cells [3]. Further, given our data showing that hyperactivation of Wnt activity by HDACis at least partially mediates the effects of those agents on CRC apoptosis [1,2], it is important to understand the specific roles of CBP- versus p300-mediated Wnt activity in HDACi-induced CRC cell apoptosis. Cells will be treated as described above, and cell proliferation will be assayed by the CellTiter cell viability/proliferation assays. In selected experiments, growth potential will also be assayed by clonogenic assays, performed as previously described [1]. Apoptotic analyses will be performed using the Vybrant Apoptosis Assay Kit #2 or the Annexin V-PE Apoptosis Detection Kit I, as previously described [12,24]. Alternatively, the Caspase-Glo luciferase system will be used to quantitate early (caspase 9 activity) and late (caspase 3 activity) apoptotic events. In addition, the extent of CRC cell differentiation will be measured by alkaline phosphatase activity, essentially as described [68].

Our hypothesis is that the effects of HDACis on promoting apoptosis of CRC cells by the hyperactivation of Wnt signaling is dependent upon the specific induction of either CBP- or p300-mediated Wnt signaling, and that by specifically blocking CBP- or p300-mediated Wnt signaling with the appropriate small molecule inhibitor, we will also suppress the induction of apoptosis by the HDACis. To the extent that induction of Wnt activity by HDACis influences other aspects of colonic cell physiology (eg, cell proliferation or differentiation), these phenomena would be specifically dependent upon CBP- or p300-mediated Wnt signaling as well. These findings, particularly, with respect to apoptosis, will have significant chemopreventive and/or therapeutic significance, because modulation of CBP/p300-mediated Wnt signaling may evolve into therapeutic approaches to induce CRC cell apoptosis, either alone or in conjunction with stimulation of excessive Wnt activity by HDACis.

We will also cotreat selected CRC cell lines with combinations of the small molecule agents (ICG-001 and IQ-1, as well as ICG-001 and ICG-427) in the presence and absence of butyrate. For selected experiments, we will use vorinostat, to compare the findings with that obtained with butyrate. As described above, the ICG-001 and IQ-1 combination will inhibit CBP-mediated Wnt activity while simultaneously preventing the cells from switching to p300-mediated signaling. For all three aims, this will be an important control to distinguish effects of diminished CBP-mediated Wnt activity from that of lower CBP– and higher p300–mediated Wnt signaling. The combination of ICG-001 and ICG-427 will simultaneously block both CBP- and p300-mediated Wnt activity. These combinations will measure the extent that signaling from both CBP and p300 pathways is required for endogenous and butyrate-stimulated Wnt activity in CRC cells. These data will be confirmed by knockdown of CBP and p300 with siRNA. The objective of these latter experiments is to determine the extent that basal and HDACi-induced Wnt activity is mediated through CBP and p300, and evaluate if significant levels of CBP/p300-independent Wnt activity occur in these cell lines. If we demonstrate that significant Wnt signaling occurs even in the absence of CBP and p300 activity, we will ascertain whether CBP- or p300-mediated Wnt signaling is required for the proapoptotic action of the HDACi butyrate in CRC cells. The combinatorial treatments will be repeated, and apoptosis measured as described above.

HDACis also influence the levels of, and activity of, p300 and CBP. For example, in Hela cells, butyrate induces the degradation of p300 through the 26S proteosome pathway [69]; in addition, butyrate can activate p300 HAT activity [69] in intestinal epithelial cells [69-71]. However, it is uncertain whether HAT activity is required to mediate the action of CBP/p300 on Wnt activity [56-59]. If HAT activity is in fact required to mediate the effects of CBP and p300 on Wnt activity, the ability of HDACis to enhance net acetylation may synergize with HAT factors to achieve these effects. Thus, HDACis may have secondary effects on HAT factors that need to be determined in order to properly interpret the findings of this proposal. Butyrate may enhance p300 degradation in Hela cells; therefore, we will ascertain the levels of CBP and p300 in the HDACi-treated CRC cell lines, using Western blot analyses and the appropriate antibodies for CBP and p300. The role of HAT activity in the modulation of Wnt activity by CBP and p300 has not yet been fully determined; however, given the possibility that changes in HAT activity induced by HDACis may influence CBP- and p300-mediated Wnt signaling, we will determine the effects of HDACis on CBP- and p300-specific HAT activity. CRC cells will be treated with HDACis, or left untreated, and CBP or p300 will be immutnoprecipitated from cell lysates with the appropriate antibodies. We will then use colorimetric or fluorescent HAT assays to determine changes of CBP and p300 HAT activity from the lysates of HDACi-treated CRC cells. These HAT assays will confirm that the MSI phenotype cell lines HCT-116 and DLD-1 express functional CBP and p300, as reported [60-63 and refs therein]. In addition, it has been reported that HDACis increase p300 autoacetylation which enhances the association of that p300 with other factors [72]. 

Therefore, using an anti-acetyl-CBP/p300 antibody, we will ascertain the effects of HDACi treatment on CBP/p300 autoacetylation in our cell lines. This will determine if there is a correlation between HAT factor autoacetylation and the ability of these factors to mediate the effects of HDACis on Wnt activity [3-6].t p300/CBP-associated factor (PCAF) activity may mediate some of the effects of CBP/p300 Wnt activity through stabilization of beta-catenin and upregulation of Wnt activity. The steady-state levels of beta-catenin and Tcf4 in the CRC cells will be evaluated by Western blot analyses subsequent to treatment with the small molecular inhibitors and the HDACis. If the small molecular inhibitors modulate levels of beta-catenin, association of PCAF with beta-catenin will be measured through coimmunoprecipitation assay. If the any of the small molecule inhibitors alters the association of PCAF with beta-catenin, siRNA-mediated knockout of PCAF will be used to determine whether CBP/p300-mediated Wnt signaling is at least partially mediated through PCAF association with beta-catenin. If so, it is expected that repression of PCAF expression and activity will repress levels of Wnt activity controlled by the action of CBP and p300. If PCAF is related to CBP/p300-mediated Wnt activity, we hypothesize that PCAF will be associated with CBP-mediated Wnt activity, because previous findings suggest that higher levels of Wnt activity in CRC cells is more dependent on CBP, compared with p300, action [3]. Thus, knockdown of PCAF would be expected to inhibit CBP-mediated Wnt activity and enhance the repression of that activity by the small molecule inhibitor ICG-001.

#### Interpretation of Results

For Aim 1, we expect to find that cells with higher levels of Wnt activity, and higher levels of induction of Wnt activity by HDACis, will exhibit a greater proportion of CBP-beta-catenin complexes compared with p300-beta-catenin complexes. If this expected result is observed, the major findings of Aim 1 would be correlated as follows. The greater increase in Wnt activity (measured by luciferase reporter assays) and apoptosis (measured by the Annexin V-PE Apoptosis Detection Kit I or the Caspase-Glo luciferase system) achieved in HWA cells treated with HDACis will be associated with a greater degree of CBP-beta-catenin complex formation (measured by co-immunoprecipitation) compared with LWA cells. Conversely, the weaker response of LWA cells to HDACis is expected to be correlated to a relatively higher association of p300 to beta-catenin compared to CBP-beta-catenin complexes. ICG-001, which interferes with the association of CBP and beta-catenin would therefore be expected to strongly suppress the marked upregulation of Wnt activity and apoptosis observed in HDACi-treated HWA cells; it is expected that ICG-001 and HDACi cotreatment would result in the following set of results: inhibition of CBP-beta-catenin complex formation, increased p300-beta-catenin complex formation, lower levels of Wnt signaling, and lower levels of apoptosis.

The weak induction of Wnt signaling and apoptosis by HDACis in LWA cells would be relatively unaffected by ICG-001, because the LWA cells are expected to have minimal CBP Wnt activity even in the absence of ICG-001. However, treatment of HWA cells with HDACis and ICG-427/IQ-1, which inhibit p300 signaling and potentiate CBP- mediated Wnt activity, would be expected to enhance the induction of Wnt activity and apoptosis by HDACis; this would be correlated with an increase in CBP-beta-catenin complexes and a decrease in p300-beta-catenin complexes. It is possible that inhibition of p300-mediated Wnt activity by ICG-427/IQ-1 may enhance the sensitivity of LWA cells to HDACis by increasing CBP-beta-catenin complex formation and action. SW620 cells, which exhibit a phenotype midway between that of HWA and LWA cells, would also be expected to exhibit moderate upregulation of CBP-beta-catenin complex formation and activity after exposure to HDACis, and would be also expected to exhibit a moderate response to the small molecule inhibitors. These expected results would be generally consistent with our preliminary data, which showed that expression of a dominant negative form of p300 significantly enhanced Wnt activity in SW620 CRC cells, possibly by shifting Wnt complexes to CBP-mediated activity. Increased Wnt activity can be reasonably expected to be associated with enhanced levels of BCT complexes, and we have found that HDACis can enhance BCT complex formation in some CRC cell lines [1,2]. We also expect that the inhibition of CBP-mediated Wnt activity will suppress the hyperactivation of Wnt activity in CRC cells treated with HDACis.

However, the interactions between HDACis and the CBP and p300 signaling pathways are likely to be complex because (1) HDACis such as butyrate stimulate CRC cell differentiation and apoptosis, which is more consistent with the p300 Wnt pathway, (2) CBP-mediated Wnt activity is likely to be correlated with higher expression of survivin, cyclinD1, and EphB4 [3,67], which is expected to favor proliferation and suppress apoptosis, while paradoxically (3) the hyperactivation of Wnt activity induced by HDACis is more likely associated with CBP-mediated Wnt activity, because blockade of CBP-Wnt signaling by ICG-001 reduces overall Wnt activity in CRC cells [3]. Therefore, the relationship between CBP/p300 signaling and the induction of Wnt activity by HDACis cannot be accurately predicted and will be determined empirically. For example, if p300- mediated Wnt activity, and not CBP-mediated activity, is responsible for the marked upregulation of Wnt activity and apoptosis in HDACi-treated HWA cells, then the expected correlations described above would be reversed. Thus, for example, treatment of HWA CRC cells with HDACis would upregulate p300-beta-catenin complex formation, Wnt activity and apoptosis, and possibly downregulate levels of survivin, cyclinD1, and EphB4, which require CBP activity for expression. If the effects of HDACis on CRC cells are dependent on p300-mediated Wnt signaling, then it is reasonable to expect the induction of Wnt activity and apoptosis by HDACis would be suppressed by ICG-427 and potentiated by ICG-001.

### Aim 2. Determination of the Role of CBP and p300 in the Maintenance of High Wnt and Low Wnt Fractions in CRC Cell Lines

The existence of high- and low-Wnt fractions in CRC cells in culture is a stable and reproducible phenomenon [1], which may be related to the known heterogeneity of in vivo tumors with respect to distribution of nuclear beta-catenin and, hence, of Wnt activity, likely of clinical significance [54,55]. It is important to distinguish high and low Wnt fractions within CRC cells (Aim 2) from the existence of HWA and LWA cell lines (Aim 1). The distinction between HWA and LWA cell lines, studied in Aim 1, evaluates the relative response of the total cell population to HDACis, with respect to induction of Wnt activity and apoptosis. In contrast, high- and low-Wnt fractions, studied in Aim 2, evaluate the different subpopulations that exist within each cell line. Thus, for example, Aim 1 is focused on evaluating the role of CBP/p300-mediated Wnt activity in the response of HWA HCT-116 cells compared with LWA HT-29 cells; Aim 2 focuses upon the role of CBP/p300-mediated Wnt signaling in maintaining the existence of separate high- and low-Wnt fractions within the HCT-116 cell population. Our hypothesis is that high- and low-Wnt cell fractions within single CRC cell lines in vitro are characterized by distinctive profiles of relative CBP- or p300-mediated Wnt activity, and that disruption of CBP/p300-mediated Wnt activity by the small molecule inhibitors ICG-001, ICG-427, or IQ-1 will significantly alter the distribution of high- and low-Wnt fractions characteristic of defined CRC cell lines.

Flow cytometry will be used to separate HCT-116 cells into their constituent high- and low-Wnt fractions in the presence or absence of butyrate [1]. This cell line exhibits a marked upregulation of the proportion of high-Wnt activity cells after exposure to the HDACi butyrate [1]. The green fluorescent protein vectors EGFP-TOP and EGFP-FOP, with wild- and mutant-type Wnt-responsive promoters will be transfected into the cells. Transfections will be performed with 20 μg of EGFP-TOP or EGFP-FOP (10-cm dishes). In some cases, to confirm that the sorted fractions do in fact differ in levels of Wnt activity, cells will be cotransfected with 4 μg of the Wnt reporter vectors LEF-OT or LEF-OF and 0.8 ng of pRLnull, a fraction of the sorted cells will be lysed in passive lysis buffer, and luciferase expression measured as described above. Six hours after transfection, cells will be harvested by scraping and transferred to 15-cm dishes. Twenty-four hours after transfection, cells will be treated with 5 mM NaB or 10 μM vorinostat, or left untreated, and collected 24 hours later for analyses.

Flow cytometric sorting of HCT-116 cells into high (“Wnt positive”) and low (“Wnt negative”) Wnt fractions based upon Wnt-specific GFP expression will be performed essentially as described [1,2]. Cells will be harvested, their density will be adjusted to 5 × 10^6^ cells per milliliter of alpha-minimal essential medium (αMEM), and samples analyzed by flow cytometry based upon fluorescence. Representative plots of relative cell numbers (counts) versus fluorescence in EGFP-TOP and EGFP-FOP transfected HCT-116 cells will be overlaid. The relative levels of CBP-beta-catenin and p300-beta-catenin complexes in each of the cell fractions will be ascertained through coimmunoprecipitation; the association of CBP and p300 with Tcf1 and Tcf4, as well as the formation of BCT complexes, will be similarly ascertained. Subsequently, the original mixed-cell populations will be cotreated with the HDACis and ICG-001, ICG-427, and/or IQ-1 (eg, ICG-001 and IQ-1 together), or with each of the small molecule inhibitors alone, followed by separation by flow sorting to determine if the proportion of cells in each fraction is altered when the CBP- or p300-mediated Wnt activity is repressed. The association of CBP and p300 with beta-catenin, Tcf1, and Tcf4, as well as the formation of BCT complexes, will be determined by coimmunoprecipitation assays in these cotreated cells. Levels of Wnt activity in the cell fractions will be ascertained using the OT/OF reporter system; we will calculate the Wnt fraction metrics described shown in Table 1 (Preliminary Studies) to determine if modulation of CBP or p300 activity alters Wnt activity per cell and/or the fraction of cells exhibiting Wnt activity [1,2]. Levels of cell proliferation, apoptosis, and differentiation in the fractions will be measured as described above.

In Table 1. T/F measures total Wnt activity in the cell population, determined by transfection with TOPFlash or FOPFlash luciferase reporter. To determine the percent Wnt positive cells (%W), cells were transfected with EGFP-TOP or EGFP- FOP vectors, and processed by flow cytometry as described in to obtain overlay plots of relative cell numbers versus fluorescence in EGFP-TOP and EGFP-FOP transfected cells. Cells with Wnt activity are those defined by high fluorescence present only in EGFP-TOP and not in EGFP- FOP samples. T/F/W is the T/F ratio divided by the percent Wnt positive cells, which provides a relative determination of Wnt activity on a per Wnt positive cell percentage basis. No NaB represents measurements of untreated cells, while NaB represents measurements after 24-hour exposure to 5 mM NaB. ΔT/F is the fold-upregulation of total Wnt activity after treatment with NaB (NaB/No NaB), Δ%W is the fold-upregulation of the percent Wnt positive cells after NaB treatment, and ΔT/F/W is the fold increase in the T/F/W ratio resulting from exposure to NaB.

**Table 1:** Upregulation of Wnt activity in NaB-treated CRC cells occurs by both an increase in the percent cells with Wnt activity and the levels of Wnt activity per cell.

	No NaB			NaB			NaB/No NaB		
Cell line	T/F	% W	T/F/W	T/F	%W	T/F/W	ΔT/F	Δ%W	ΔT/F/W
HCT-116	4.8	9.8	0.49	104.6	46.0	2.3	21.8	4.7	4.7
DLD-1	6.2	10.0	0.62	95.5	20.5	4.7	15.4	2.1	7.6
LS174T	14.5	19.8	0.73	193.6	40.4	4.8	13.3	2.0	6.6
LoVo	5.7	11.7	0.49	17.9	16.5	1.1	3.2	1.4	2.2
SW48	14.7	26.4	0.56	32.7	34.6	0.95	2.2	1.3	1.7

#### Interpretation of Results

We expect that CBP-mediated Wnt activity will be associated with the high-Wnt fraction in untreated cells, and p300-mediated Wnt activity with the low-Wnt fraction. If so, the correlated findings will be similar to that described in Aim 1; the high-Wnt fraction of the cell population will be associated with higher levels of CBP-beta-catenin complexes, Wnt activity, and sensitivity to HDACis. However, as explained in Aim 1, it is possible that the induction of apoptosis through hyperactivated Wnt activity may occur through either CBP- or p300- mediated Wnt signaling, which needs to be determined empirically. Therefore, it is difficult to predict which of the two Wnt coactivators will be predominantly involved in changes in the proportion of high and low Wnt fractions induced by HDACis. This will be determined empirically, through the experiments outlined in Aim 2. Discovery of an association between CBP- or p300-mediated Wnt activity and the maintenance of high- and low-Wnt fractions in CRC cells in vitro would suggest the possibility of modulating CBP- and/or p300-mediated Wnt activity in vivo, to either repress Wnt signaling and proliferation at the invasive front of CRC tumors, which constitutes a high Wnt fraction component of the neoplasm, or in conjunction with HDACis, hyperactivate Wnt signaling in the high-Wnt activity fraction at the tumor’s invasive front, in order to target these potentially metastatic cells [57,58] for apoptosis [1,2]. Therefore, understanding the roles of CBP- and p300-mediated Wnt signaling in intra-CRC heterogeneity, particularly, heterogeneity of Wnt activity, is expected to lead to therapeutic approaches targeting the more invasive and metastatic components of CRCs.

### Aim 3. In Vitro Determination of the Role of CBP and p300 Wnt Modulation in the Progression of Colonic Neoplasia In Vitro

Aim 1 of the current proposal focuses upon the role of CBP- and p300-mediated Wnt activity for the upregulation of Wnt signaling and apoptosis in CRC cells treated with HDACis; the major objective of Aim 2 is to determine the role of CBP/p300-mediated Wnt signaling in the maintenance, and response to HDACis, of high- and low-Wnt fractions within CRC cell populations. Thus, after determining the activity of CBP- and p300-mediated Wnt activity, and response to HDACis, between (Aim 1) and within (Aim 2) established carcinoma cell lines, Aim 3 extends these findings to evaluate CBP- and p300-mediated Wnt activity across four colonic cell lines ranging from normal to metastatic and thus representing an in vitro model of tumorigenic progression, and as a mechanism by which the tumorigenic phenotype of colonic cells can be modulated by altering the relative levels of CBP- versus p300-mediated Wnt signaling.

Therefore, to contrast and compare the effects of CBP- and p300-mediated Wnt activity on colonic neoplastic progression (normal cells to adenoma to carcinoma to metastasis), we will use, as an in vitro model system, the normal colon cell line CCD-841CoN, the adenoma line LT97 [7,8], as well as the primary colon carcinoma cell line SW480 and the lymph node metastasis cell line SW620, which was derived from the same patient as SW480 cells. The LT97 cell line is of especial use for these experiments; the LT97 human adenoma cell line was isolated from a microadenoma [7], the earliest possible neoplasm that can be surgically isolated, from a human patient with hereditary familial adenomatous polyposis (FAP). Therefore, as expected, LT97 cells are characterized by the presence of C-terminus truncated APC protein and a lack of full-length, wild-type protein [7,8]. While we expect LT97 cells to exhibit both endogenous Wnt activity as well as significant upregulation of this activity by HDACis, a recent study has suggested that early APC mutant adenomas may not exhibit nuclear beta-catenin and Wnt signaling, which occurs later in later adenomas and in progression to carcinoma [73]. Thus, the level of Wnt signaling in LT97 cells has not been formally documented, and this determination, which is important in and of itself, with be a component of Aim 3.

Unlike colon carcinoma cells, LT97 cells are incapable of growing in soft agar [3]; the LT97 line exhibits an early premalignant phenotype, quite distinct from that of the typical carcinoma cell lines used as in vitro models of CRC. Thus, LT97 cells are a unique model of the early stages of APC mutation-initiated human colonic tumorigenesis [7]. Based upon both our findings on the effects of HDACis on CRC cells and on the literature, we believe that butyrate, and hence, dietary fiber, would be more effective in suppressing CRC during the earlier stages of tumorigenesis. Consistent with this, it has been shown that the LT97 cells are more sensitive to the growth-suppressing effects of butyrate than are CRC HT-29 cells [8]. Likewise, the nonmetastatic SW480 cell line [74] exhibits greater sensitivity to the effects of butyrate on Wnt activity and apoptosis than the metastatic cell line SW620, derived from the same patient [1,2]. This suggests that early-stage neoplastic colonic cells are more sensitive to HDACis than their later-stage counterparts. The CCD-841Con normal colonic cell line is also of particular interest for this project; our collaborators have observed that while the CBP-Wnt inhibitor ICG-001 induced caspase activity, indicative of apoptosis, in SW480 and HCT-116 CRC cells, it did not do so in the CCD-841CoN line [3]. Therefore, there is a clearly demonstrated difference between the normal colonic line CCD-841CoN and CRC cell lines in the physiological response to ICG-001. Thus suggests differences between normal and neoplastic CRC cells in CBP-Wnt-mediated signaling and downstream phenotypic effects; differences in the association between CBP/p300-Wnt activity and cellular phenotype will be a fundamental focus of Aim 3 of this proposal. Therefore, we hypothesize that (1) the four cell lines to be tested in Aim 3 differ in their relative levels of CBP- versus p300-mediated Wnt activity, and (2) these differences in CBP/p300-Wnt activity influence the phenotypic characteristics of these cell lines, characteristics associated with each cell line’s position along the pathway of neoplastic progression.

The first part of Aim 3 is to delineate the phenotypic characteristics of the four cell lines. SW480, SW620 cells and LT97 cells will be cultured as previously described [1,2,7]. CCD-841CoN cells will be cultured as described in the relevant information sheet available from the American Type Culture Collection. Cell growth/proliferation, differentiation, and apoptosis will be measured as described above; the effects of the HDACis butyrate and vorinostat on these processes will also be measured as described above. Growth in soft agar will be measured as previously described [7]. We will also confirm that culture of normal CCD-841CoN cells and LT97 microadenoma cells in the culture medium typically used for CRC cell lines (ie, αMEM plus 10% fetal bovine serum) will result in significantly reduced cell proliferation and viability, demonstrating a dependence of these less tumorigenic cells on additional growth factors. Wnt activity, in the presence and absence of HDACis, will be measured through the TOP Flash/FOPFlash reporter assay as well as the accumulation of active, dephosphorylated beta-catenin [1,2]. These measurements will form the core set of evaluations with respect to phenotypic differences in cell physiology and Wnt activity between the four cell lines studied in Aim 3.

Coimmunoprecipitation will be used to determine the levels of CBP- and p300-beta-catenin complexes in these four cell lines in the presence and absence of the HDACis, treated as described above. The association of CBP and p300 with Tcf1 and Tcf4, as well as the formation of BCT complexes will also be measured by coimmunoprecipitation assays. Cells will be cotreated with HDACis (butyrate or vorinostat, as described above) and with ICG-001, ICG-427, and/or IQ-1 (eg, ICG-001 and IQ-1 together), or with each of the small molecule agents alone, and the levels of Wnt activity, apoptosis, proliferation, differentiation, CBP- or p300-beta-catenin binding, and expression of survivin, c-myc, cyclin D1, EphB2, and EphB4 will be measured as described above. The same coimmunoprecipitation experiments will be performed to ascertain if the formation of complexes between CBP and p300 and Tcf proteins are altered in these cell lines. The levels of Wnt signaling present in each cell line will be measured using the luciferase reporter system. Repression of Wnt activity by one of the small molecule inhibitors would provide information as to whether the Wnt activity present in the cells is predominantly CBP- or p300-mediated, as explained previously. For example, repression by ICG-001 would specifically repress CBP-mediated Wnt activity; ICG-427 would specifically repress p300-mediated Wnt activity. In addition, determination of growth in soft agar will be performed as described [7], to determine whether tumorigenicity can be altered through modulation of CBP- and p300-specific Wnt transcriptional programs. Therefore, we will be correlating the phenotypic measurements of progression and tumorigenicity with (1) complex formation between CBP or p300 and beta-catenin and Tcfs, (2) Wnt activity as evaluated by reporter assays, and (3) expression of survivin, c-myc, cyclin D1, EphB2, and EphB4.

Consistent with our underlying hypothesis for Aim 3, we expect to observe differences between the four cell lines in the relative levels of CBP- versus p300-mediated Wnt activity, both in the presence and absence of HDACis. Thus, the relative levels of CBP–beta-catenin complexes versus p300–beta-catenin complexes will differ between the four cell lines, as will effects of the small molecule inhibitors on both endogenous- and HDACi-stimulated Wnt activity. For example, one of the cell lines may exhibit relatively higher levels of CBP–beta-catenin complexes compared with the other lines; it would be expected that the Wnt activity present in this cell line would be sensitive to, and repressed by, ICG-001, because most of the Wnt signaling in this cell line would be CBP mediated. In contrast, another cell line showing relatively higher levels of p300–beta-catenin complexes would exhibit greater sensitivity to the Wnt activity-repressing effects of ICG-427, which targets p300-mediated signaling. Differential sensitivity of the cell lines to HDACis, with respect to CBP/p300-Wnt activity, would also be revealed by treatment with the small molecule inhibitors. For example, if ICG-001, but not ICG-427, represses induction of Wnt activity usually observed by treatment of a cell line with HDACis, that finding would support the hypothesis that HDACis predominantly influence CBP-mediated, but not p300-mediated, Wnt activity in those colonic cells.

Therefore, one possibility is that as cells progress along the neoplastic continuum, CBP-mediated Wnt activity will predominate over p300-mediated activity, which is consistent with the finding that the CBP-Wnt inhibitor ICG-001 specifically inhibits the growth of neoplastic as opposed to normal colonic cells [3]. This is also consistent with increased expression of the CBP-Wnt target EphB4, which is associated with more advanced tumorigenic phenotypes [67]. Thus, it is reasonable to assume both enhanced CBP-Wnt activity and EphB4 expression (and, possibly, decreased EphB2 levels) comparing cells with increasing neoplastic phenotypes (eg, CCD-841Con to LT97 to SW480 to SW620).

However, the greater sensitivity of LT97 adenoma cells to butyrate compared with carcinoma cell lines [8], suggests the possibility that changes in the relative levels of CBP- and p300-mediated Wnt activity may favor CBP-Wnt activity in the earlier stages of colonic carcinogenesis. Thus, phenotypic differences between cell types representative of phases in colonic neoplastic progression may be altered by CBP/p300-mediated Wnt activity. If so, we will attempt to use modulation of the levels of CBP- and p300-mediated Wnt activity as a tool to alter the phenotype of LT97 cells in the direction of the less tumorigenic normal cell line CCD-841CoN or the more tumorigenic CRC cell line SW480. Both CCD-841CoN normal colonic cells and SW480 CRC cells have been shown by our collaborators to significantly differ in their phenotypic response to ICG-001, with SW480, but not CCD-841CoN, cells showing enhanced apoptosis when treated with this agent. This approach will determine whether modulation of CBP- and p300-mediated Wnt activity can transition an early colonic neoplasm (represented by LT97 cells) to phenotypes with greater or lesser tumorigenicity. The strategy will use molecular and pharmacological tools to shift the ratio of CBP- versus p300-mediated Wnt activity of LT97 cells toward that observed in CCD-112CoN or SW480 cells, as ascertained by the experiments described above. This will be achieved using the small molecule agents ICG-001, ICG-427, or IQ-1, as well as expression vectors for CBP, p300, and DN300, and, if required, siRNA for CBP and p300. While expression vectors and siRNA may be used as secondary modulators of CBP and p300 activity, in all cases at least one of the small molecule inhibitors will be used, so as to modify Wnt-specific CBP- and p300-mediated gene expression and consequent cellular phenotypes. For example, while DN300 is expected to inhibit the totality of p300 activity, IQ-427 will specifically disrupt Wnt-specific p300 activity.

Concentrations of agents and treatment length will be determined empirically, until a defined phenotypic change is observed. We will measure Wnt signaling, apoptosis and apoptotic response to HDACis, proliferation and the growth suppressing effects of HDACis, differentiation, as well as growth in soft agar, as described above. We will also determine whether LT97 cells altered in the direction of a SW480-like phenotype are able to efficiently proliferate in the same cell culture medium as CRC cells. An endpoint for the process of phenotypic transition will be established; that is, a time point will be determined at which point any observed phenotypic modification of LT97 cells will be considered as “complete.” For example, acquisition of the ability to grow in soft agar or to proliferate independent of a specialized culture medium, would be suggestive of transition to a more tumorigenic, SW480 cell-like phenotype. Further, enhanced sensitivity to the growth suppressive and apoptosis-inducing effects of HDACis such as butyrate are also suggestive of a more advanced tumorigenic phenotype. Conversely, we will have established phenotypic differences distinguishing CCD-841Con cells from LT97 cells (see above), and evaluation of these metrics will be used to ascertain if CBP- or p300-signaling modifications transition LT97 cells toward a more normal, CCD-841CoN-like cellular phenotype. Our hypothesis, consistent to our expectations in the first part of Aim 3, is that each of the four cell lines is characterized by its own profile of CBP- versus p300-mediated Wnt activity, and that forcing LT97 cells to exhibit a CBP/p300 Wnt profile of CCD-841CoN or SW480 cells will promote LT97 cells to exhibit specific phenotypic characteristics of those cell lines, respectively.

Dependent upon the differences that are observed between the four cell types, we expect that modulation of CBP/p300-mediated Wnt activity of LT97 cells, toward either that characteristic of CCD-841CoN cells or SW480 cells, will shift the LT97 cellular phenotype in those two directions, respectively. Once we have established a CBP/p300-mediated treatment regimen that is able to transition LT97 cells to lesser or greater tumorigenicity, we will conduct microarray analyses to determine the global changes in gene expression responsible for these CBP- or p300-mediated changes. LT97 cells will be treated with the particular combination of agents found to alter cell phenotype in the required directions (toward the CCD-841Con and SW480 phenotypes), or left untreated. Wnt signaling, apoptosis, and apoptotic response to HDACis, proliferation, differentiation, as well as growth in soft agar will be measured as described above. Measurements of the expression of survivin, cyclinD1, c-myc, EphB4, and EphB2 will also be performed; we would expect that transition of LT97 cells to a more tumorigenic phenotype would be characterized by increased expression of the first four genes and decreased expression of EphB2; this pattern would be expected to be reversed with transition of LT97 cells to a more normal colonic phenotype. However, this expectation is dependent upon whether a more tumorigenic phenotype is associated with increased CBP-mediated Wnt activity (see above).

At the defined time point at which phenotypic transition is considered complete, mRNA will be isolated with Oligotex mRNA Direct Kit. Genus Biosystems will perform the microarray processing and data analyses for these experiments, using the Agilent human whole genome (41,000+ human genes) oligo microarray. Targeted genes will be validated by Northern blot analyses or quantitative reverse transcription polymerase chain reaction (RT-PCR). A small set of selected discovered genes that are reasonably seen as major candidates for mediating the observed changes in cellular phenotype, particularly those genes that are known to be Wnt pathway targets, will be further analyzed. These three microarray measurements (original LT97, CCD-112CoN-like LT97 and SW480-like LT97) will be repeated eight times to yield statistically significant quantitative data on changes in gene expression underlying the observed changes in cellular phenotype.

We will determine, by Western blot analyses, whether expression of these genes are modulated by treatment of LT97 cells by the small molecule inhibitors; further, using DN-Tcf4 to block Wnt activity, we will determine whether expression of these selected genes are directly or indirectly dependent upon Wnt signaling. The expression of selected genes will then be directly up- or downregulated through overexpression (expression vectors) or knockdown (siRNA) in order to recapitulate the phenotype changes resulting from the treatments (small molecule inhibitors, expression vectors/siRNA for CBP or p300) above. If changes in the expression of specific CBP/p300 Wnt target genes can mimic phenotypic changes induced by modulation of CBP- or p300-mediated Wnt signaling, this will strongly suggest that the relevant genes are downstream effectors of CBP- or p300-mediated Wnt signaling, and are at least partially responsible for the changes in cellular phenotype induced by the CBP- or p300-targeted treatments.

#### Interpretation of Results

We expect that LT97 cells will exhibit Wnt activity and modulation of this activity by HDACis, consistent with the general literature; however, this would be inconsistent with the controversial possibility that the earliest stages of colonic initiation do not exhibit Wnt activity [69]. We also expect to identify a subset of CBP- or p300-target genes that differ in expression between the original LT97 cells and the LT97 cells with modified phenotypes, and that subsequent directed modulation of selected genes will at least partially recapitulate the changes in LT97 cell phenotype induced by the methods for up- or downregulating CBP/p300-mediated Wnt activity. Given that CCD-841Con cells were shown to be relatively insensitive to the apoptosis-inducing properties of the CBP-Wnt inhibitor ICG-001 [3], it is possible that those cells already have sharply downregulated CBP-Wnt signaling. For example, it has been shown that inhibiting CBP-Wnt activity by CBP siRNA represses the ability of ICG-001 to downregulate CBP-Wnt signaling. This repression is due to the fact that most of the downregulation of CBP-Wnt activity had already occurred due to the siRNA [3]. By analogy, if CBP-Wnt signaling is naturally low in normal colonic cells, it is reasonable to expect that an inhibitor of that signaling will have little or no effect [3]. Therefore, it is possible that inhibition of CBP-Wnt activity to very low levels is associated with the CCD-841CoN phenotype; if so, a combination of ICG-001 and CBP siRNA, perhaps coupled with overexpression of p300, would be the optimal treatment regimen for transitioning LT97 cells to a more CCD-841CoN phenotype. In this case, the ICG-001 and CBP siRNA would both repress CBP-Wnt signaling and promote p300-Wnt signaling; overexpression of p300 would perhaps synergize to facilitate the switch from CBP- to p300-mediated Wnt activity. On the other hand, the promotion of CBP-Wnt activity over p300-Wnt activity may be more effective in inducing a more tumorigenic phenotype in LT97 cells.

The initial evaluation of functional significance of identified genes will be performed as Aim 3 of the current proposal, and more in depth analyses of physiological relevance of these genes will be incorporated into future projects that will build upon the findings of the present study. Future studies will also determine whether observed differences in gene expression are observed in a broad spectrum of CRC cell lines, characterized by different Wnt pathway activating mutations. The development of treatment regimens that can either promote or inhibit tumorigenesis of early stage colonic neoplasms, and the determination of the patterns of gene expression that mediate these changes in tumorigenic potential, may lead to novel chemopreventive and therapeutic approaches against CRC. Thus, for example, a treatment regimen identified that induces LT97 cells to transition to a more CCD-841Con phenotype can serve as the basis for in vivo studies aimed at developing CBP/p300-targeted therapeutics effective at suppressing colonic tumorigenesis, reversing early stage tumorigenesis, or treating established CRC.

### Research Design: Pitfalls and Alternative Approaches

We have extensive experience with the methodologies to be used in this proposal. The potential pitfalls for the specific aims, and alternative approaches that will be used, is outlined as follows. Aim 1: Some effects of HDACis on CRC cell physiology are independent of the modulation of Wnt activity. Wnt-mediated effects of HDACis, working through CBP/p300, may be obscured by opposing non-Wnt effects of HDACis. If necessary, CRC cells stably transfected with an inducible form of DN-Tcf4, which represses Wnt activity, can be used to separate Wnt-specific and non-Wnt-specific effects of HDACis on cell physiology [1,2]. The repression of Wnt activity in DN-Tcf4 transfected cells can be used to ascertain the proportion of HDAC-induced apoptosis mediated by Wnt activity [1,2], including CBP- and p300-specific effects. It is possible, although unlikely, that we will observe that neither blockade of CBP-Wnt activity (ICG-001] or of p300-Wnt activity (ICG-427] influences the upregulation of Wnt activity and of apoptosis by HDACis. If this occurs, the results of the ICG-001/IQ-1 and ICG-001/ICG-427 combinatorial cotreatments will be further analyzed. In the unlikely event that HWA HCT-116 and DLD-1 cells do not exhibit CBP and p300 HAT activity due to their MSI status, which would be inconsistent with previous reports [3,60-63 and refs therein], we will substitute these cells with the HWA Colo201 cell line, which does not exhibit the MSI phenotype [60-63 and refs therein]. However, if HCT-116 and DLD-1 cells do exhibit, as expected, CBP and p300 activity, they are preferable to Colo201 cells because (1) Colo201 cells are semiadherent and more difficult to culture and transfect, (2) our collaborators have already established that HCT-116 cells are sensitive to the proapoptotic action of ICG-001, and (3) it is useful to evaluate the role of CBP/p300-mediated Wnt signaling in the action of HDACis in MSI-positive cells, such as HCT-116 and DLD-1, compared with non-MSI cells such as SW480 and SW620. Aim 2: CRC cell lines transfected with inducible DN-Tcf4 will be used if the findings suggest that effects of HDACis on cellular physiology (eg, apoptosis) that are independent of Wnt activity are masking the Wnt-dependent effects measured here. If so, the DN-Tcf4 cell lines can be used to determine the effects of non-Wnt-mediated events through effective suppression of endogenous and HDACi activated Wnt activity as described in Aim 1 [1]. In the unlikely event that the MSI status of HCT-116 cells makes them unsuitable for the experiments of Aim 2, they will be substituted with the SW480 cell line. Aim 3: It is possible that LT97 cells will not exhibit Wnt activity, consistent with a recent report suggesting a lack of nuclear beta-catenin in early stage APC mutant neoplasms, but inconsistent with the predominant view in the literature that activated Wnt signaling is the earliest initiating event in colonic tumorigenesis. If this unexpected finding is observed, we will change the focus of Aim 3 from modulating CBP/300-mediated Wnt activity in LT97 cells to altering that activity in SW480 cells in order to transition these cells to a more (SW620) tumorigenic or less (LT97) tumorigenic phenotype, respectively. Therefore, if LT97 cells do not exhibit the expected endogenous Wnt activity, we will use SW480 cells as the model system for CBP/p300-Wnt induced changes in cellular phenotype, instead of the LT97 cell line. All experiments will be as outlined above, with the exception of the change in cell type. We fully expect that the well characterized, Wnt-positive SW480 cell line to effectively represent a suitable model system for the experiments of Aim 3, because these cells lie in between LT97 and SW620 cells in the continuum of colonic neoplastic progression, and the Wnt signaling pathway in these cells has been studied in detail in our laboratory. However, we believe that LT97 cells, if they exhibit Wnt activity, would represent a more effective model given that they are more sensitive to HDACis and are derived from an earlier stage of tumorigenesis that may be more amenable to treatment. However, the SW480 cell line represents a reasonable and effective alternative for the successful completion of Aim 3, if the LT97 cells prove unsuitable for these experiments. Assuming that LT97 cells exhibit Wnt activity as we expect, is possible that we will be unable to transition LT97 cell phenotypes into those more similar to CCD-841CoN and SW480 cells or that we will be unable to identify the gene expression profiles associated with any observed phenotypic changes. If so, we will change the objectives of the second half of Aim 3 and instead examine the patterns of CBP/p300-Wnt-mediated gene expression altered by treatment of colonic cells with HDACis. Our collaborators have already demonstrated that SW480 CRC, but not normal colonic, cells exhibit Wnt-mediated changes in cell physiology upon treatment with ICG-001, an agent which specifically disrupts interactions between CBP and beta-catenin and inhibits Wnt transcriptional activity [3]. Thus, SW4840 CRC and CCD-841CoN normal colonic clearly exhibit differences in CBP/p300-mediated Wnt signaling. Further, it is likely that HDACis, which preferentially induce apoptosis in neoplastic cells and upregulate Wnt activity [1,2], will modulate CBP/p300 signaling differently in SW480 and CCD-841CoN cells. Thus, this approach represents a reasonable alterative for the second part of Aim 3. However, if we are successful in modifying LT97 cell phenotypes and in identifying the CBP/p300- mediated Wnt targeted gene expression profiles responsible for the altered phenotypes, then the experiments outlined in the preceding paragraph will be performed as part of future, more in depth studies designed to determine the patterns of gene expression modulated by CBP and p300 throughout the entire process of colonic neoplasia, with an emphasis on those phenotypically relevant genes whose expression is modulated by HDACis.

### Statistics

Student’s *t*-test will be used to determine statistical significance (*P*<.05). We have successfully used this biostatistical method to evaluate data from our previous studies on Wnt signaling that were generated by experimental methodologies identical to, or similar to, those proposed in for the current project [1,2]. When appropriate, normality of data will be ascertained using the Kolmogorov-Smirnov test (with Dallal-Wilkinson-Lilliefor *P* values), the D’Agostino and Pearson test, or the Shapiro-Wilk test. Choice of normality test will depend upon sample, size; in most cases, Kolmogorov-Smirnov will be used. In the event that the data do not follow a normal distribution, the nonparametric Mann-Whitney U test will be used.

## Results

Research on this proposal is ongoing. To date, findings from this proposal, which cover all three specific aims, have been published in the Journal of Cancer [61,62,63]. Thus, we have shown that CBP and p300 activities influence butyrate-mediated Wnt hyperactivation, that CBP activity is absolutely required for efficient hyperactivation of Wnt activity by butyrate, that the maintenance of high-Wnt activity cell fractions requires CBP-mediated Wnt activity, and that there are significant cell-type differences among neoplastic colonic cell lines, including early stage LT97 microadenoma cells, in the manner in which CBP versus p300 activity influences Wnt signaling and colonic cell physiology. Experiments are ongoing.

## Discussion

This manuscript describes a R15 AREA grant proposal funded by the National Institutes of Health (National Cancer Institute) upon initial submission. Our findings with respect to this research [61,62,63] clearly demonstrate that CBP- and p300-mediated Wnt activities affect Wnt signaling hyperactivation, and influence apoptosis and proliferation of neoplastic colonic cells; further, CBP and p300 differ in how they affect these processes, and these differences likely influence colonic neoplastic initiation and progression.

The ultimate objective of this line of research, and its clinical relevancy, is to determine whether modulators of CBP/p300-mediated Wnt signaling, in combination with HDACis such as butyrate and the FDA, vorinostat, exert more efficacious antineoplastic effects against CRC than the small molecules or HDACis alone. We therefore envision the possible use of combinatorial treatment with HDACis and small molecule inhibitors of CBP/p300-mediated Wnt signaling in the role of chemoprevention (eg, dietary fiber/butyrate) of therapy to suppress tumor progression and/or as novel chemotherapeutic agents against advanced disease. This possibility would be advanced by in vivo studies demonstrating that specific combinations of HDACis and modulators of CBP/p300-Wnt activity result in marked suppression of intestinal tumorigenesis in mouse models of human CRC. Future in vitro experiments will be aimed at dissecting the molecular mechanisms by which CBP and p300 modulate Wnt activity, and promote cell differentiation and apoptosis, in a large range of CRC cell lines. Further, we will also dissect the mechanisms, whereby HDACis influence CBP/p300-mediated Wnt activity. More in depth microarray analyses will be used to determine the full genetic programs up- or downregulated by HDACis through the CBP- or p300-mediated Wnt signaling, and identify downstream targets of CBP-Wnt versus p300-Wnt signaling. Further, we propose that mouse models of CRC initiation and progression, such as the Cre-Lox model of APC inactivation [74] be used to ascertain the in vivo chemopreventive efficacy of cotreatment with HDACis and ICG-001, ICG-427, and/or IQ-1, compared with these agents used in isolation. Because this mouse model can progress to carcinoma, it can be used to determine the role of CBP versus p300 Wnt signaling in the in vivo progression from normal cell to adenoma to carcinoma. Analyses of primary cells derived from human patients will be used to determine the relationship between relative levels of CBP- or p300-beta-catenin complexes and the expression of genes that are targets of CBP- versus p300-mediated Wnt activity. These studies will strengthen the clinical relevancy of our initial findings, to determine whether combinatorial use of HDACis with modulators of CBP/p300-mediated Wnt signaling represents an effective chemopreventive and therapeutic strategy against CRC. Therefore, the findings of the proposed study have the potential to lead to novel pharmacological agents that can enhance the antineoplastic action of HDACis for CRC chemoprevention and/or therapy.

## Multimedia Appendix 1

This is the funded grant review.

## Multimedia Appendix 2

This is the funded grant award letter.

## Abbreviations

αMEM: alpha-minimal essential medium
APC: adenomatous polyposis coli
BCT: Beta-catenin-Tcf
CBP: CREB binding protein
CRC: colorectal cancer
CREB: cAMP response element binding
DN-Tcf4: dominant negative Tcf4
ESCs: embryonic stem cells
FAP: familial adenomatous polyposis
FDA: US Food and Drug Administration
GSK-3beta: glycogen synthase kinase-3 beta
HAT: histone acetyl transferase
HDACis: histone deacetylase inhibitors
HWA: high-fold induction of Wnt activity and apoptosis
LWA: low-fold induction of Wnt activity and apoptosis
MSI: microsatellite instability
MSS: microsatellite stable
NaB: sodium butyrate
PCAF: p300/CBP-associated factor
RT-PCR: reverse transcription polymerase chain reaction
SAHA: suberoylanilide hydroxamic acid
siRNA: small-interfering RNA
Tcf/Lef: transcription factor/lymphoid enhancer-binding factor
TSA: trichostatin A
