# Supplementary material for: Determination of the Role of CBP- and p300-Mediated Wnt Signaling on Colonic Cells
Source: JMIR Res Protoc. 2016 May 13;5(2):e66. doi: 10.2196/resprot.5495 (PMC4884266; doi:10.2196/resprot.5495)
Supplement: Supplementary file 1 [file resprot_v5i2e66_app1.pdf]

SUMMARY STATEMENT
(Privileged Communication)

PROGRAM CONTACT:
Elizabeth Woodhouse
301-435-1878
elisa@mail.nih.gov

Release Date: **10/07/2009**

Application Number: **1 R15 CA149589-01**

Principal Investigator

BORDONARO, MICHAEL PHD

Applicant Organization: THE COMMONWEALTH MEDICAL COLLEGE

Review Group: **CAMP**
Cancer Molecular Pathobiology Study Section

Meeting Date: **09/17/2009**
Council: **JAN 2010**
Requested Start: **04/01/2010**

RFA/PA: **PA06-042**
PCC: **F4TB**
Dual PCC: **NLM DUAL**
Dual IC(s): **DK**

Project Title: **Determination of the role of CBP and p300 mediated Wnt signaling on colonic cells**

SRG Action: **Impact/Priority Score: 26**
Human Subjects: **10-No human subjects involved**
Animal Subjects: **10-No live vertebrate animals involved for competing appl.**

Project Year	Direct Costs Requested	Estimated Total Cost
1	150,000	150,000
TOTAL	150,000	150,000

ADMINISTRATIVE BUDGET NOTE: The budget shown is the requested budget and has not been adjusted to reflect any recommendations made by reviewers. If an award is planned, the costs will be calculated by Institute grants management staff based on the recommendations outlined below in the COMMITTEE BUDGET RECOMMENDATIONS section.

NOTE TO APPLICANT: A new scoring system is in use for NIH grant applications [(<http://grants.nih.gov/grants/guide/notice-files/NOT-OD-09-024.html> and <http://grants.nih.gov/grants/guide/notice-files/NOT-OD-09-025.html>)] and requires new percentile bases. Some of the new percentile bases for the January, 2010 council cycle will not be calculated until October 23, 2009. If your application is eligible for percentiling, but none is currently shown, that percentile will be available in the eRA Commons after October 23, 2009.

1R15CA149589-01 BORDONARO, MICHAEL

RESUME AND SUMMARY OF DISCUSSION: The overall goal of this proposal is to further explore the contribution of the Wnt signaling pathway to colorectal cancer (CRC) by exploring the interactions between β-catenin and the transcriptional coactivators CREB binding proteins (CBP) and p300. Results have potential to identify targets in the Wnt pathway that could be exploited for therapeutics or prevention of CRC. Strengths include: an interesting concept under consideration, fairly well designed experiments, the use of CPB and P300 selective inhibitors, and the applicant's expertise in Wnt signaling. Some reviewer's expressed concern with the descriptive nature of the studies and that the selected cell lines were less than optimal. However, most felt that the experimental design could provide new information on the mechanisms of drug inhibitors for Wnt. Additionally, it was found to be perfect to introduce students to cancer research. The strong intuitional support for the AREA objectives, the opportunity to include both graduate and undergraduate students from all three academic centers, the commitment and support from Dr. Flynn, Deputy Director, MBR Cancer Center, and a well established and successful cancer researcher, far outweigh the concerns raised. Following discussion it was concurred that the applicant is a new investigator committed to the training of young scientist and that the proposed studies will enhance the research program of The Commonwealth Medical College, a newly established medical school. The overall strengths were score driving for the study section.

DESCRIPTION (provided by applicant): The Wnt signaling pathway, mediated through active beta-catenin, is responsible for initiating the majority of cases of human colorectal cancer (CRC), and we have previously shown that hyper-activation of this pathway by histone deacetylase inhibitors (HDACis), such as butyrate, can induce the death of CRC cells. An important cellular switch that mediates the effects of Wnt signaling activation is variation in the association between beta-catenin and the transcriptional coactivators CREB binding protein (CBP) and p300. Association of CBP with beta-catenin is thought to activate a set of genes linked to cell proliferation, while the p300-mediated Wnt genetic program is believed to promote cell differentiation. Small molecule agents have been discovered that modulate CBP/p300 Wnt transcriptional programs by altering the association of CBP and p300 to beta-catenin. ICG-001 and ICG-427 inhibit CBP and p300 mediated Wnt activity, respectively, while IQ-1 prevents the shift from CBP-mediated to a p300-mediated Wnt activity. Aim 1 of this proposal is designed to determine the role of CBP and p300 mediated Wnt signaling in the response of CRC cells to HDACis. Cells will be cotreated with HDACis and ICG-001, ICG-427, or IQ-1 and the levels of Wnt activity, apoptosis, proliferation, differentiation, and CBP- or p300-beta-catenin binding measured. Aim 2 of this proposal is to determine the role of CBP/p300-mediated Wnt activity in the maintenance of high- and low-Wnt fractions in single CRC cell populations, which may mirror similar heterogeneity observed in human tumors and which may be of clinical significance. Aim 3 will compare the effects of CBP and p300 mediated Wnt activity on CRC initiation and progression, utilizing CRC cell line model systems of initiation and progression: the normal colon cell lines CCD-841CoN, the adenoma line LT97, the primary colon carcinoma cell line SW480, and the lymph node metastasis cell line SW620. Cells will be treated with HDACis and the small molecule agents, and assayed as described above. We will also attempt to use changes in CBP and p300 mediated Wnt signaling to shift colonic cells between cell type, modifying CBP and p300 mediated gene expression in the LT97 adenoma line to shift the adenoma phenotype to more characteristic of the CCD- 841CoN normal cells, or the SW480 carcinoma cells. We will utilize microarray analyses to determine the patterns of gene expression responsible for these CBP or p300 mediated changes in colonic neoplastic phenotype. The findings generated from this study will lead to future, more in-depth projects to further dissect the action of CBP/p300 Wnt-mediated transcriptional programs in colonic neoplasia, with an emphasis on methods to modulate these genetic programs for chemopreventive effect.

PUBLIC HEALTH RELEVANCE: The Wnt signaling pathway is responsible for initiating the majority of cases of human colorectal cancer, and we have previously shown that hyper-activation of this pathway by histone deacetylase inhibitors (HDACis) can induce the death of colorectal cancer cells. Wnt signaling mediated by the proteins CBP or p300 differentially influence decisions of cancer cell growth, differentiation, or death, and small molecule agents exist that can specifically block CBP and p300 mediated Wnt signaling. Through the use of these small molecule inhibitors, we will determine how CBP and p300 influence colonic tumorigenesis; the findings of this study can be utilized to devise methodologies, targeting CBP and p300 mediated Wnt activity, to prevent the development and progression of human colorectal cancer, decreasing the morbidity and mortality associated with this disease.

CRITIQUE 1:

Significance: 2
Investigator(s): 2
Innovation: 4
Approach: 3
Environment: 2

Overall Impact:

Strengths

- The research proposed is considered highly significant for understanding contribution of WNT pathway components and inhibitors thereof to malignant transformation and prevention.
- A series of defined inhibitors of the pathway in a well defined set of cell lines would generate interesting new insights into early and late transformation events in colonic epithelial cells.
- Inclusion of new undergraduate and graduate students into the research proposed is planned and feasible.
- Preliminary data from the PI’s work and collaborators’ work support the hypotheses and feasibility.

Weaknesses

- No major weaknesses are perceived for the application under this grant mechanism (AREA)

1. Significance:

Strengths

- The proposal addresses a significant question in CRC initiation, progression and signaling via the WNT pathway.
- The proposal uses a series of known inhibitors with characteristics defined earlier by others (HDACi’s, WNT interaction protein inhibitors).
- The proposal also builds on previous experience, publications and preliminary data from the applicant that include use of a well-defined set of colorectal normal, early and late transformation cells.
- In addition, the applicant uses expertise in previous studies with heterogeneity in cultured cells to sketch out a series of interesting experiments.

- The in vitro phenotypic characterizations of cell lines are appropriate and the inhibitor studies could lend support to further in vivo studies plus broader applications.

Weaknesses

- The series of studies is completely focused on in vitro characteristics of cell lines which may be sufficient for the current scope of the proposal, but should be considered only the first step in to characterization of malignant transformation.
- Much of these studies are correlative and will not yet address contributions of particular drivers at the molecular level or the contribution of distinct posttranslational modifications.

2. Investigator(s):

Strengths

- Dr. Bordonaro has good expertise and training in the field of study.
- Highly qualified to carry out studies.
- The collaborator is well chosen.

Weaknesses

- None noted.

3. Innovation:

Strengths

- Some innovative use of cell lines of different transformation state and drug combinations to interrogate the wnt pathway.

Weaknesses

- Uses mostly standard technology to characterize phenotypes.

4. Approach:

Strengths

- In vitro cell biology approaches are a well established and the inhibitors used are well characterized. Also, the cell lines that are proposed for the experiments represent a good choice and are well rationalized.
- The proposal should generate interesting new data and has the potential to find new avenues under the third aim as well as in the drug combinations proposed.
- Signal transduction studies focus on protein:protein interactions

Weaknesses

- Cell biology approaches are mostly proposed, and there is little consideration for post-translational modifications that might be relevant.

5. Environment:

Strengths

- Appropriate for a new school that has not been a recipient of NIH support, and this would strengthen the research environment.
- Dr. Flynn, the associate Dean for research is an experienced scientist and his expertise from his previous position at the Cancer Center in Morgantown WVA is great strength to guide this project and AREA.

Weaknesses

- None noted.

Protections for Human Subjects:

Not Applicable.

Vertebrate Animals:

Not Applicable.

Biohazards:

- No concerns.

Budget and Period of Support:

Recommend as Requested

CRITIQUE 2:

Significance: 3
Investigator(s): 4
Innovation: 4
Approach: 4
Environment: 4

Overall Impact:

Strengths

- The proposed experiments will provide support for a new investigator at a new Institution that will clearly strengthen the research environment of The Commonwealth Medical College and The University of Scranton and Marywood University.
- Many of the very straightforward experiments in the specific aims are amenable to undergraduate and graduate student training, allowing students to test reasonably developed hypotheses in a meritorious research environment.

Weaknesses

- The overall focus is on cell lines with questionable relationship to human colorectal carcinoma.
- Histone deacetylase inhibitors have pleiotropic effects. Thus, the proposed studies have limited potential to make a major impact on treatment or prevention of colorectal carcinoma.

1. Significance:

Strengths

- The experiments are likely to provide insights into how CPB and p300 independently influence Wnt activity, and how their expression regulates proliferation, differentiation and apoptosis of colon cancer cells.
- Use of novel, relatively selective inhibitors of CPB and P300, may provide insights toward strategies for chemoprevention and therapy of colorectal carcinoma.

Weaknesses

- Strategies using combinations of inhibitors (as many as three in some experiments) are likely to yield complex results that dampen the significance of specific effects of CPB and P300 on wnt expression and subsequent biologic endpoints.
- Lack of determination of or interpretation of quantitative differences (e.g. in differences in factors studied to co-immunoprecipitate; potential differences in activation of Wnt targets) dampens the significance of the results to be obtained.

2. Investigator(s):

Strengths

- The application is submitted by a new investigator who is well trained and has several publications on Wnt signaling, thus appears qualified to direct the proposed studies.

Weaknesses

- The applicant does not have an outstanding track record in publications, with most in modest impact factor Journals.
- As a new investigator in his first independent position, the applicant has limited experience in student training.

3. Innovation:

Strengths

- The hypothesis that differences in association of CBP and p300 might regulate the Wnt activity and determine whether proliferation, differentiation, or apoptosis occur is an innovative concept in the field of colon cancer research.

Weaknesses

- Approaches are standard and not innovative; some of the cell line models used are archaic and lack any innovation as a model for colon tumors.

4. Approach:

Strengths

- Specific aim 2, which sorts high from low Wnt activity fractions is very attractive, as results can be determined from an isogenic background and will not be influenced by the considerable genetic differences among cell lines in aim 1.

Weaknesses

- The applicant has exhaustively considered alternative possibilities for p300/CPB regulation and Wnt activity. Unfortunately, testing each of the alternative possibilities of Wnt regulation could be

very time consuming, and what is not considered is alternative Wnt-independent mechanisms by which agents that have a plethora of effects, such as butyrate, are likely to have on biologic endpoints (e.g. proliferation, differentiation, apoptosis). It is unclear how biologic effects (e.g. apoptosis) that are independent of Wnt activity and are “masking” the Wnt effects will be determined.

- It is highly unlikely that high and low Wnt activity cells can be maintained in vitro.
- cDNA array analyses are premature, especially for a three year R15.

5. Environment:

Strengths

- It is difficult to assess the environment of a new Institution, but numerous letters suggest that sufficient core faculties are available for this project as well as financial support for the applicant, and local Universities appear eager to send students to The Commonwealth Medical College for training. Thus the environment seems conducive to fulfilling the goals of this R15 application.

Weaknesses

- There appears to be a lack of a core of senior investigators to provide guidance for the new/young faculty such as this applicant.

Protections for Human Subjects:

- Not Applicable

Vertebrate Animals:

- Not Applicable

Biohazards:

- No concerns.

Budget and Period of Support:

Recommend as Requested

Additional Comments to Applicant:

- The writing is often very dense, especially when the numerous alternatives in Wnt activation are considered.
- Simple diagrams demonstrating the applicant’s best predictions of the expected results of the inhibitors alone and in combination would clarify the proposal
- In addition, for reasons noted in evaluation of the applicant, above, inclusion of a senior scientist would strengthen the application.

CRITIQUE 3:

Significance: 5

Investigator(s): 5
Innovation: 6
Approach: 6
Environment: 2

Overall Impact:

Strengths

- There is great confidence that this application will achieve the R15 goals of strengthening the TCMC research environment and exposing undergraduate, graduate, and medical students to research.
- Thus, from the standpoint of the goals of the R15 granting mechanism, this is an excellent application.

Weaknesses

- The track record of the applicant, both in terms of publications and training, was seen as a weakness.

1. Significance:

Strengths

- Understanding the molecular mechanism for variable sensitivities of CRC cells to HDACis could have important clinical implications.
- Pharmacologic approaches have the additional advantage of being closer to the clinic than other approaches.

Weaknesses

- The approach has a number of major and minor weaknesses, and hence the impact of these studies on colon cancer is unclear.

2. Investigator(s):

Strengths

- The applicant has published papers regarding butyrate and Wnt signaling over the last 7+ years, particularly in colon cancer, which is the topic of this grant application.
- Real support from a number of key players and the effort the applicant has devoted to establish this research program indicate that there is a bona fide effort to implement this research.

Weaknesses

- The applicant was previously an Associate Research Scientist in the Department of Pharmacology at Yale University from 1998-2006 and 2007-2008, resulting in ~4 first author primary research papers.
- The applicant has had limited experience mentoring students.
- Collectively these were considered to be moderate weaknesses.

3. Innovation:

Strengths

- The concept that extreme (high or low) levels of Wnt signaling lead to apoptosis, and further, that HDACi lead to elevated Wnt signaling that may sensitize cells to apoptosis.

Weaknesses

- The approaches do not mechanistically address they hypothesis rigorously; the inclusion of more genetic approaches would increase enthusiasm. This was seen as a moderate weakness that detracted from the novel hypothesis.

4. Approach:

Strengths

- Based on the applicant's published and preliminary data that there is a correlation with apoptosis and high Wnt activity induced by multiple HDACis in a panel of 10 CRC cell lines the applicant will test if HDACi treatment of *i*) high (HWA) versus low (LWA) CRC cell lines, *ii*) FACS sorted populations of the same cell line, *iii*) four colonic derived cell lines representing progressively more malignant phenotypes or *iv*) changes the association of p300 or CBP with beta-catenin or changes Wnt activity or apoptosis upon exposure to small molecules that selectively inhibit p300 HAT activity (ICG-427) or CBP HAT activity (ICG-001) or PP2A (IQ-1).
- Additionally, genomic signatures will be determined on a cell line. The applicant is well equipped to undertake these studies; they utilize multiple settings to test the same issue, and should provide some insight into the variable effects of HDACis.

Weaknesses

- The approach on the whole relies solely on pharmacological intervention to dissect the role of CBP and p300 in the variable effect of HDACi on CRC cell lines, which is seen as a major weakness.
- HDACis will have very broad effects above and beyond the Wnt pathway, which is conceptually not really taken into account.

5. Environment:

Strengths

- TCMC appears to be an ideal environment for R15 funding.
- Students will be drawn into the program not only from TCMC itself, but also from the University of Scranton and Marywood University, as evidenced from the letters of collaboration.
- TCMC has pledged to cover the salary of undergraduate students during the summer.
- TCMC has pledged support for research, providing the applicant with a startup package including capital equipment, as well as some infrastructure, for example high performance cluster computing (HPCC) site, a Waters HPLC system, *etc*.
- The fact that the applicant has sought out support and garnered letters to draw in new students was viewed very positively.
- Environment was the driving force in the positive score.

Weaknesses

- Research infrastructure is still in development, for example one -80 freezer is listed as common equipment for 28 labs, and a number of key core facilities are contracted from private companies.
- At this point neither TCMC nor the applicant have an established strong track record in biomedical research.

Protections for Human Subjects:

Not Applicable.

Vertebrate Animals:

Not Applicable

Budget and Period of Support:

Recommend as Requested

THE FOLLOWING RESUME SECTIONS WERE PREPARED BY THE SCIENTIFIC REVIEW OFFICER TO SUMMARIZE THE OUTCOME OF DISCUSSIONS OF THE REVIEW COMMITTEE ON THE FOLLOWING ISSUES:

COMMITTEE BUDGET RECOMMENDATIONS: The budget was recommended as requested.

NOTICE: In 2008 NIH modified its policy regarding the receipt of resubmission (formerly termed amended) applications. Detailed information can be found by accessing the following URL address: http://grants.nih.gov/grants/policy/amendedapps.htm

MEETING ROSTER

Cancer Molecular Pathobiology Study Section
Oncology 1-Basic Translational Integrated Review Group
CENTER FOR SCIENTIFIC REVIEW
CAMP
September 17, 2009 - September 18, 2009

CHAIRPERSON
PRIVES, CAROL , PHD
PROFESSOR
DEPARTMENT OF BIOLOGICAL SCIENCES
GRADUATE SCHOOL OF ARTS AND SCIENCES
COLUMBIA UNIVERSITY
NEW YORK, NY 10027

MEMBERS
ABOUNADER, ROGER , PHD
ASSOCIATE PROFESSOR OF NEUROLOGY
DEPARTMENTS OF NEUROLOGY AND MICROBIOLOGY
UNIVERSITY OF VIRGINA HEALTH SYSTEM
CHARLOTTESVILLE, VA 22908

ABRAMS, JOHN MICHAEL, PHD *
ASSOCIATE PROFESSOR
DEPARTMENT OF CELL BIOLOGY
UNIVERSITY OF TEXAS SOUTHWESTERN
MEDICAL CENTER
DALLAS, TX 75390

BAND, VIMLA , PHD
PROFESSOR AND VICE CHAIR OF RESEARCH
DEPARTMENT OF GENETICS, CELL BIOLOGY AND
ANATOMY
ASSOCIATE DIRECTOR OF BREAST CANCER CENTER
EPPLEY CANCER CENTER
UNIVERSITY OF NEBRASKA MEDICAL SCHOOL
OMAHA, NE 68198

BERBERICH, STEVEN J., PHD
PROFESSOR AND INTERIM CHAIR
DEPARTMENT OF BIOCHEMISTRY AND MOLECULAR
BIOLOGY
SCHOOL OF MEDICINE
WRIGHT STATE UNIVERSITY
DAYTON, OH 45435

BOISE, LAWRENCE H., PHD *
PROFESSOR
DEPARTMENT OF HEMATOLOGY AND MEDICAL
ONCOLOGY
WINSHIP CANCER INSTITUTE
ATLANTA, GA 30322

CALDWELL, CHARLES W., MD, PHD
PROFESSOR AND DIRECTOR
DEPARTMENT OF PATHOLOGY AND
ANATOMICAL SCIENCES
ELLIS FISCHEL CANCER CENTER
UNIVERSITY OF MISSOURI-COLUMBIA
COLUMBIA, MO 65203

CHALFANT, CHARLES E., PHD
ASSOCIATE PROFESSOR
DEPARTMENT OF BIOCHEMISTRY
SCHOOL OF MEDICINE
VIRGINIA COMMONWEALTH UNIVERSITY
RICHMOND, VA 23298

CHEN, JIANDONG , PHD
PROFESSOR
DEPARTMENT OF INTERDISCIPLINARY ONCOLOGY
H. LEE MOFFITT CANCER CENTER
TAMPA, FL 33612

CHEN-KIANG, SELINA Y., PHD *
PROFESSOR
DEPARTMENT OF PATHOLOGY
WEILL MEDICAL COLLEGE OF CORNELL UNIVERSITY
NEW YORK, NY 10021

COREY, SETH JOEL, MD *
PROFESSOR
CHILDREN'S MEMORIAL HOSPITAL
ROBERT H. LURIE COMPREHENSIVE CANCER CENTER
FEINBERG SCHOOL OF MEDICINE
NORTHWESTERN UNIVERSITY
CHICAGO, IL 60611

COUNTER, CHRISTOPHER M., PHD
ASSOCIATE PROFESSOR
DEPARTMENTS OF PHARMACOLOGY
AND CANCER BIOLOGY
DUKE UNIVERSITY MEDICAL CENTER
DURHAM, NC 27710

CRAMER, SCOTT D., PHD *
ASSOCIATE PROFESSOR
DEPARTMENT OF CANCER BIOLOGY
WAKE FOREST UNIVERSITY SCHOOL OF MEDICINE
WINSTON-SALEM, NC 27157

GAIANO, NICHOLAS R., PHD *
PROFESSOR
INSTITUTE FOR CELL ENGINEERING
JOHNS HOPKINS UNIVERSITY SCHOOL OF MEDICINE
BALTIMORE, MD 21205

GALLICK, GARY E., PHD *
PROFESSOR
DEPARTMENT OF CANCER BIOLOGY
M.D. ANDERSON CANCER CENTER
UNIVERSITY OF TEXAS
HOUSTON, TX 77030

GOLEMIS, ERICA A., PHD
SENIOR MEMBER
INSTITUTE FOR CANCER RESEARCH
FOX CHASE CANCER CENTER
PHILADELPHIA, PA 19111

GOODELL, MARGARET A., PHD
PROFESSOR
DEPARTMENTS OF PEDIATRICS, MOLECULAR
AND HUMAN GENETICS AND IMMUNOLOGY
BAYLOR COLLEGE OF MEDICINE
HOUSTON, TX 77030

HEMENWAY, CHARLES S., MD, PHD *
ASSOCIATE PROFESSOR
DEPARTMENT OF PEDIATRICS
STRITCH SCHOOL OF MEDICINE
LOYOLA UNIVERSITY CHICAGO
MAYWOOD, IL 60153

HUANG, SUYUN MD, PHD *
ASSOCIATE PROFESSOR
DEPARTMENT OF NEUROSURGERY
UNIVERSITY OF TEXAS M.D. ANDERSON CANCER
CENTER
HOUSTON, TX 77030

JERRY, D. JOSEPH, PHD *
ASSOCIATE PROFESSOR
DEPARTMENT OF VETERINARY AND ANIMAL SCIENCE
COLLEGE OF FOOD AND NATURAL RESOURCES
UNIVERSITY OF MASSACHUSETTS
AMHERST, MA 01003

JOHNSON, DEBORAH , PHD
PROFESSOR
DEPARTMENT OF BIOCHEMISTRY AND MOLECULAR
BIOLOGY
KECK SCHOOL OF MEDICINE
UNIVERSITY OF SOUTHERN CALIFORNIA
LOS ANGELES, CA 90033

KNUDSEN, KAREN E., PHD
ASSOCIATE PROFESSOR
DEPARTMENT OF CANCER BIOLOGY
KIMMEL CANCER CENTER
THOMAS JEFFERSON UNIVERSITY
PHILADELPHIA, PA 19107

LAND, HARTMUT , PHD
ROBERT AND DOROTHY MARKIN PROFESSOR AND
CHAIR
DEPARTMENTS OF BIOMEDICAL GENETICS,
ONCOLOGY, BIOCHEMISTRY AND BIOPHYSICS
UNIVERSITY OF ROCHESTER MEDICAL CENTER
ROCHESTER, NY 14642

LEES, JACQUELINE A., PHD
PROFESSOR
DAVID H. KOCH INSTITUTE FOR INTEGRATIVE
CANCER RESEARCH
MASSACHUSETTS INSTITUTE OF TECHNOLOGY
CAMBRIDGE, MA 02139

LOU, ZHENKUN , PHD *
ASSISTANT PROFESSOR
DEPARTMENT OF ONCOLOGY
MAYO CLINIC COLLEGE OF MEDICINE, ROCHESTER
ROCHESTER, MN 55905

LOWY, DOUGLAS R., MD
DEPUTY DIRECTOR
CENTER FOR CANCER RESEARCH
NATIONAL CANCER INSTITUTE
NATIONAL INSTITUTES OF HEALTH
BETHESDA, MD 20892

MUGANDA, PERPETUA M., PHD
PROFESSOR
DEPARTMENT OF BIOLOGY
COLLEGE OF ARTS AND SCIENCES
NORTH CAROLINA A&T STATE UNIVERSITY
GREENSBORO, NC 27284

QUELLE, DAWN E., PHD
ASSOCIATE PROFESSOR
DEPARTMENT OF PHARMACOLOGY
COLLEGE OF MEDICINE
UNIVERSITY OF IOWA
IOWA CITY, IA 52242

REED, STEVEN I., PHD *
PROFESSOR
DEPARTMENT OF MOLECULAR BIOLOGY
THE SCRIPPS RESEARCH INSTITUTE
LA JOLLA, CA 92037

RICHARDSON, CHRISTINE A., PHD *
ASSOCIATE PROFESSOR
DEPARTMENT OF BIOLOGY
UNIVERSITY OF NORTH CAROLINA AT CHARLOTTE
CHARLOTTE, NC 28223

RICHMOND, ANN , PHD *
PROFESSOR AND VICE CHAIR
DEPARTMENT OF CANCER BIOLOGY
VANDERBILT UNIVERSITY SCHOOL OF MEDICINE
NASHVILLE, TN 37232

SHIH, LE-MING , MD, PHD
PROFESSOR
DEPARTMENT OF PATHOLOGY
JOHNS HOPKINS UNIVERSITY SCHOOL OF MEDICINE
BALTIMORE, MD 21231

SHILATIFARD, ALI , PHD *
INVESTIGATOR
STOWERS INSTITUTE FOR MEDICAL RESEARCH
KANSAS CITY, MO 64110

THORBURN, ANDREW M., PHD
PROFESSOR
DEPARTMENT OF PHARMACOLOGY
SCHOOL OF MEDICINE
CENTER AT FITZSIMMONS
UNIVERSITY OF COLORADO HEALTH SCIENCE
AURORA, CO 80045

WALENSKY, LOREN D., MD, PHD *
ASSISTANT PROFESSOR
DEPARTMENT OF CANCER IMMUNOLOGY AND AIDS
DANA-FARBER CANCER INSTITUTE
BOSTON, MA 02115

WANG, HONG-GANG , PHD
LOIS HIGH BERSTLER PROFESSOR
DEPARTMENT OF PHARMACOLOGY
COLLEGE OF MEDICINE
PENNSYLVANIA STATE UNIVERSITY
HERSHEY, PA 17033

WANG, ZHOU , PHD *
PROFESSOR
DEPARTMENT OF UROLOGY
UNIVERSITY OF PITTSBURGH
PITTSBURGH, PA 15232

WEISSMAN, BERNARD E., PHD *
PROFESSOR
DEPARTMENTS OF PATHOLOGY AND LABORATORY MEDICINE AND PEDIATRICS
LINEBERGER COMPREHENSIVE CANCER CENTER
UNIVERSITY OF NORTH CAROLINA
CHAPEL HILL, NC 27599

WELLSTEIN, ANTON , MD, PHD
PROFESSOR
DEPARTMENT OF ONCOLOGY
LOMBARDI CANCER CENTER
GEORGETOWN UNIVERSITY
WASHINGTON, DC 20057

WONG, ALBERT J., MD *
PROFESSOR
DEPARTMENT OF NEUROSURGERY
STANFORD UNIVERSITY
STANFORD, CA 94305

ZHANG, DONG-ER , PHD
PROFESSOR
DEPARTMENT OF PATHOLOGY
MOORES UCSD CANCER CENTER
UNIVERSITY OF CALIFORNIA SAN DIEGO
LA JOLLA, CA 92093

SCIENTIFIC REVIEW ADMINISTRATOR

SIERRA-RIVERA, ELAINE , PHD
SCIENTIFIC REVIEW OFFICER
CENTER FOR SCIENTIFIC REVIEW
NATIONAL INSTITUTES OF HEALTH
BETHESDA, MD 208927804

GRANTS TECHNICAL ASSISTANT

MCALPINE, TAKISHA
EXTRAMURAL SUPPORT ASSISTANT
CENTER FOR SCIENTIFIC REVIEW
NATIONAL INSTITUTE OF HEALTH
BETHESDA, MD 20892

* Temporary Member. For grant applications, temporary members may participate in the entire meeting or may review only selected applications as needed.

Consultants are required to absent themselves from the room during the review of any application if their presence would constitute or appear to constitute a conflict of interest.
