# Supplementary material for: Determination of the Role of CBP- and p300-Mediated Wnt Signaling on Colonic Cells
Source: JMIR Res Protoc. 2016 May 13;5(2):e66. doi: 10.2196/resprot.5495 (PMC4884266; doi:10.2196/resprot.5495)
Supplement: Supplementary file 2 [file resprot_v5i2e66_app2.pdf]

Grant Number: 1R15CA149589-01

Principal Investigator(s):
Michael Bordonaro, PHD

Project Title: Determination of the role of CBP and p300 mediated Wnt signaling on colonic cells

Ms. Mancuso, Sandra
Director, Sponsored Programs
501 Madison Avenue
Scranton, PA 18510

Award e-mailed to: smancuso@tcmedc.org

Budget Period: 03/01/2010 – 02/28/2013
Project Period: 03/01/2010 – 02/28/2013

Dear Business Official:

The National Institutes of Health hereby awards a grant in the amount of $183,203 (see “Award Calculation” in Section I and “Terms and Conditions” in Section III) to THE COMMONWEALTH MEDICAL COLLEGE in support of the above referenced project. This award is pursuant to the authority of 42 USC 241 42 CFR 52 and is subject to the requirements of this statute and regulation and of other referenced, incorporated or attached terms and conditions.

Acceptance of this award including the “Terms and Conditions” is acknowledged by the grantee when funds are drawn down or otherwise obtained from the grant payment system.

Each publication, press release or other document that cites results from NIH grant-supported research must include an acknowledgment of NIH grant support and disclaimer such as “The project described was supported by Award Number R15CA149589 from the National Cancer Institute. The content is solely the responsibility of the authors and does not necessarily represent the official views of the National Cancer Institute or the National Institutes of Health.”

Award recipients are required to comply with the NIH Public Access Policy. This includes submission to PubMed Central (PMC), upon acceptance for publication, an electronic version of a final peer-reviewed, manuscript resulting from research supported in whole or in part, with direct costs from National Institutes of Health. The author's final peer-reviewed manuscript is defined as the final version accepted for journal publication, and includes all modifications from the publishing peer review process. For additional information, please visit http://publicaccess.nih.gov/.

Award recipients must promote objectivity in research by establishing standards to ensure that the design, conduct and reporting of research funded under NIH-funded awards are not biased by a conflicting financial interest of an Investigator. Investigator is defined as the Principal Investigator and any other person who is responsible for the design, conduct, or reporting of NIH-funded research or proposed research, including the Investigator's spouse and dependent children. Awardees must have a written administrative process to identify and manage financial conflict of interest and must inform Investigators of the conflict of interest policy and of the Investigators' responsibilities. Prior to expenditure of these awarded funds, the Awardee must report to the NIH Awarding Component the existence of a conflicting interest and within 60 days of any new conflicting interests identified after the initial report. Awardees must comply with these and all other aspects of 42 CFR Part 50, Subpart F. These requirements also apply to subgrantees, contractors, or collaborators engaged by the Awardee under this award. The NIH website http://grants.nih.gov/grants/policy/coi/index.htm provides additional information.

If you have any questions about this award, please contact the individual(s) referenced in Section IV.

NIH NGA R | Version: 385 - 11/24/2009 13:33:07 | Generated on: 03/01/2010 00:02:43

Sincerely yours,

Sean Hine
Grants Management Officer
NATIONAL CANCER INSTITUTE

Additional information follows

NIH NGA R | Version: 385 - 11/24/2009 13:33:07 | Generated on: 03/01/2010 00:02:43

SECTION I – AWARD DATA – 1R15CA149589-01

Award Calculation (U.S. Dollars)

**Federal Direct Costs**	$150,000
**Federal F&A Costs**	$33,203
**Approved Budget**	$183,203
**Federal Share**	$183,203
**TOTAL FEDERAL AWARD AMOUNT**	$183,203
**AMOUNT OF THIS ACTION (FEDERAL SHARE)**	$183,203

SUMMARY TOTALS FOR ALL YEARS		
**YR**	**THIS AWARD**	**CUMULATIVE TOTALS**
1	$183,203	$183,203

Fiscal Information:

**CFDA Number:**	93.396
**EIN:**	1260812968A1
**Document Number:**	RCA149589A
**Fiscal Year:**	2010

IC	*CAN*	*2010*
CA	8479568	$183,203

NIH Administrative Data:
PCC: F4TB / **OC**: 414A / **Processed**: HINES 02/24/2010

SECTION II – PAYMENT/HOTLINE INFORMATION – 1R15CA149589-01

For payment and HHS Office of Inspector General Hotline information, see the NIH Home Page at http://grants.nih.gov/grants/policy/awardconditions.htm

SECTION III – TERMS AND CONDITIONS – 1R15CA149589-01

This award is based on the application submitted to, and as approved by, NIH on the above-titled project and is subject to the terms and conditions incorporated either directly or by reference in the following:

a. The grant program legislation and program regulation cited in this Notice of Award.
b. Conditions on activities and expenditure of funds in other statutory requirements, such as those included in appropriations acts.
c. 45 CFR Part 74 or 45 CFR Part 92 as applicable.
d. The NIH Grants Policy Statement, including addenda in effect as of the beginning date of the budget period.
e. This award notice, INCLUDING THE TERMS AND CONDITIONS CITED BELOW.

(See NIH Home Page at 'http://grants.nih.gov/grants/policy/awardconditions.htm' for certain references cited above.)

An unobligated balance may be carried over into the next budget period without Grants Management Officer prior approval.

This grant is subject to Streamlined Noncompeting Award Procedures (SNAP).

In accordance with P.L. 110-161, compliance with the NIH Public Access Policy is now mandatory. For more information, see NOT-OD-08-033 and the Public Access website: http://publicaccess.nih.gov/.

This award represents the final year of the competitive segment for this grant. Therefore, see the NIH Grants Policy Statement (12/1/2003 version) for closeout requirements at: http://grants.nih.gov/grants/policy/nihgps_2003/NIHGPS_Part8.htm#_Toc54600151.

A final Financial Status Report (FSR) (SF 269) must be submitted through the eRA Commons (Commons) within 90 days of the expiration date; see NIH Guide Notice NOT-OD-07-078 for additional information on this electronic submission requirement. The final FSR must indicate the exact balance of unobligated funds and may not reflect any unliquidated obligations. There must be no discrepancies between the final FSR and the Payment Management System's (PMS) Federal Cash Transaction Report (SF-272).

Furthermore, unless an application for competitive renewal is submitted, additional grant closeout documents consisting of a Final Invention Statement and Certification form (HHS 568), (not applicable to training, construction, conference or cancer education grants) and a final progress report must also be submitted within 90 days of the expiration date.

NIH also strongly encourages electronic submission of the final progress report and the final invention statement through the Closeout feature in the Commons. If the final progress report and final invention statement are not submitted electronically, copies of the HHS 568 form may be downloaded at: http://grants.nih.gov/grants/forms.htm.

Submissions of the final progress report and HHS 568 may be e-mailed as PDF attachments to the NIH Central Closeout Center at: deascentralized@od.nih.gov

Paper submissions of the final progress report and the HHS 568 may be faxed to the NIH Central Closeout Center at 301-480-2304 or mailed to the NIH Central Closeout Center at the following address:

NIH/OD/OER/DEAS
Central Closeout Center
6705 Rockledge Drive, Room 2207
Bethesda, MD 20892-7987 (for regular or U.S. Postal Service Express mail)
Bethesda, MD 20817 (for other courier/express mail delivery only)

The final progress report should include, at a minimum, a summary of progress toward the achievement of the originally stated aims, a list of significant results (positive and/or negative), a list of publications and the grant number. If human subjects were included in the research, the final progress report should also address the following:

- Report on the inclusion of gender and minority study subjects (using the gender and minority Inclusion Enrollment Form as provided in the PHS 2590 and available at http://grants.nih.gov/grants/forms.htm).
- Where appropriate, indicate whether children were involved in the study or how the study was relevant for conditions affecting children (see “Public Policy Requirements and Objectives-Requirements for Inclusiveness in Research Design-Inclusion of Children as Subjects in Clinical Research” in the PHS 398 at URL http://grants.nih.gov/grants/policy/nihgps_2003/NIHGPS_Part5.htm#_Toc54600090)
- Describe any data, research materials (such as cell lines, DNA probes, animal models), protocols, software, or other information resulting from the research that is available to be shared with other investigators and how it may be accessed.

Note, if this is the final year of a competitive segment due to the transfer of the grant to another institution, then not all the requirements stated above are applicable. Specifically a Final Progress Report is not required. However, a final FSR is required and should be submitted electronically as noted above. In addition, if not already submitted, the Final Invention Statement is required and should be sent directly the assigned Grants Management Specialist.

Treatment of Program Income:
Additional Costs

SECTION IV – CA Special Terms and Conditions – 1R15CA149589-01

INFORMATION: In accordance with the National Cancer Institute's (NCI's) Fiscal Year (FY) 2010 funding policies, this award has been issued at 100% of the adjusted requested level. Future year committed levels* have been adjusted accordingly.

* committed level: The level of support calculated by applying the NCI funding plan to the corrected recommended level for each budget category for all years of the project period.

Spreadsheets used to calculate this award are available upon request.

INFORMATION: Future year total cost commitments appearing on the award notice under "Recommended Future Year Total Cost Support" have been calculated by applying the negotiated facilities and administrative cost rate(s) in effect at the time of this FY 2010 award to the committed total direct cost level for each future year.

INFORMATION This is a Modular Grant Award without direct cost categorical breakdown in accordance with guidelines published in the 12/15/98 NIH Guide for Grants and Contracts, web address: http://grants.nih.gov/grants/guide/notice-files/not98-178.html. Recipients are required to allocate and account for all costs related to this award by category within their institutional accounting system in accordance with applicable cost principles.

INFORMATION: In accordance with the Notice: NOT-OD-02-017 entitled, "GRADUATE STUDENT COMPENSATION" published on December 10, 2001, in the NIH Guide for Grants and Contracts, total direct costs (salary, fringe benefits and tuition remission) for graduate students are provided at the NIH maximum allowable amount (zero level of the Ruth L. Kirschstein National Research Service Award stipend in effect at the time of the competing award). Support recommended for future years has been adjusted accordingly, if applicable. The full guide Notice describing the level of compensation allowed for a graduate student can be found at: http://grants.nih.gov/grants/guide/notice-files/NOT-OD-02-017.html.

INFORMATION: This award, including the budget and the budget period, has been discussed between Julie Peoples of the National Cancer Institute and Sandra Mancuso on February 16, 2010.

STAFF CONTACTS

The Grants Management Specialist is responsible for the negotiation, award and administration of this project and for interpretation of Grants Administration policies and provisions. The Program Official is responsible for the scientific, programmatic and technical aspects of this project. These individuals work together in overall project administration. Prior approval requests (signed by an Authorized Organizational Representative) should be submitted in writing to the Grants Management Specialist. Requests may be made via e-mail.

Grants Management Specialist: Julie Peoples
Email: peoplesj@mail.nih.gov **Phone**: 301-496-7208 **Fax**: 301-496-8601

Program Official: Elizabeth Woodhouse
Email: elisa@mail.nih.gov **Phone**: 301-435-1878 **Fax**: 301-480-0864

SPREADSHEET SUMMARY
GRANT NUMBER: 1R15CA149589-01

INSTITUTION: THE COMMONWEALTH MEDICAL COLLEGE

Budget	***Year 1***
TOTAL FEDERAL DC	$150,000
TOTAL FEDERAL F&A	$33,203
TOTAL COST	$183,203

Facilities and Administrative Costs	***Year 1***
F&A Cost Rate 1	41%
F&A Cost Base 1	$80,982
F&A Costs 1	$33,203
